# Development of artificial neural network models to predict the PAMPA effective permeability of new, orally administered drugs active against the coronavirus SARS-CoV-2

**DOI:** 10.1007/s13721-023-00410-9

**Published:** 2023-02-06

**Authors:** Chrysoula Gousiadou, Philip Doganis, Haralambos Sarimveis

**Affiliations:** School of Chemical Engineering, National Technical University of Athens, Heroon Polytechneiou 9, 15780 Zografou, Athens, Greece

**Keywords:** COVID-19, PAMPA, Permeability, Artificial neural network, Ensemble modelling, Descriptors

## Abstract

Responding to the pandemic caused by SARS-CoV-2, the scientific community intensified efforts to provide drugs effective against the virus. To strengthen these efforts, the “COVID Moonshot” project has been accepting public suggestions for computationally triaged, synthesized, and tested molecules. The project aimed to identify molecules of low molecular weight with activity against the virus, for oral treatment. The ability of a drug to cross the intestinal cell membranes and enter circulation decisively influences its bioavailability, and hence the need to optimize permeability in the early stages of drug discovery. In our present work, as a contribution to the ongoing scientific efforts, we employed artificial neural network algorithms to develop QSAR tools for modelling the PAMPA effective permeability (passive diffusion) of orally administered drugs. We identified a set of 61 features most relevant in explaining drug cell permeability and used them to develop a stacked regression ensemble model, subsequently used to predict the permeability of molecules included in datasets made available through the COVID Moonshot project. Our model was shown to be robust and may provide a promising framework for predicting the potential permeability of molecules not yet synthesized, thus guiding the process of drug design.

**Supplementary Information:**

The online version contains supplementary material available at 10.1007/s13721-023-00410-9.

## Introduction

From the onset of the pandemic caused by the virus SARS-CoV-2, the scientific community intensified efforts to provide drugs effective against the disease COVID-19 (Ferreira and Andricopulo 2020). In this context, the COVID Moonshot project (Delft et al. 2021) was launched as a worldwide collaboration between scientists aiming to identify pre-clinical candidate molecules potent against the virus, for oral use. To date, almost 21,000 structurally diverse molecules have been submitted to the project’s website (PostEra 2022). Whilst the bioactivity of the molecules designed and submitted to the project is currently under investigation and while sub-micromolar IC_50_ has been reported for a number of them (PostEra 2022), important factors such as permeability, selectivity, pharmacokinetics, pharmacodynamics and toxicity remain to be optimized to improve their drug-like profile (Erlanson 2020). Permeability across the biological membranes decisively influences the degree of a drug’s absorption and bioavailability (Homayun et al. 2019) and is therefore routinely evaluated through high-throughput screening (HTS) in the early stages of drug design using either cell-based or cell-free permeation systems (Masungi et al. 2008; Balani et al. 2005; Berben et al. 2018).

A cell-free, low-cost and easy to handle in vitro method for rapid in vivo permeability predictions—the PAMPA assay (Parallel Artificial Membrane Permeability Assay)—was introduced in 1998 by Kansy et al. (1998). PAMPA predicts in vivo permeability only via passive diffusion and is currently a preferred HTS method in the pharmaceutical industry (Schmidt and Lynch 2022). PAMPA is successful in establishing structure–activity/structure–property relationships (SARs/SPRs) and hit-to-lead optimization due to the lack of active transport systems or metabolizing enzymes (Fortuna et al. 2007). An analytical description of the method is provided in Sect. 2 section of the manuscript.

Apart from the costly, time and effort-consuming experimental studies, *in silico* approaches like quantitative structure–activity relationship (QSAR) models are reliably used as HTS methods for hit-to-lead optimization (Chi et al. 2019; Sun et al. 2017; Oja and Maran 2015a, b, c, 2016a, b, 2018; Roy et al. 2021; Diukendjieva et al. 2019) due to the short computational time required to screen large-sized datasets and the high accuracy of the models in making correct predictions. Nevertheless, despite its advantages, the QSAR approach has a limited role in the drug design process, being mainly used in the early stages for the exclusion of molecules with a low permeability profile (Dahlgren and Lennernäs 2019). Further contribution to the drug development process is rather restricted, primarily due to the choice of the descriptors involved in the analyses that fail to provide clear insight into which structural features influence permeability. Indeed, alternate models have been reported that link different molecular descriptors to the permeation process. For the most part, the Lipinski-like physicochemical properties of molecules (e.g. lipophilicity, hydrogen bond donors and acceptors, and molecular mass) (Lipinski 2000; Lipinski et al. 2001) and charge-related surface area descriptors were successfully related to permeability (Chi et al. 2019; Sun et al. 2017; Oja and Maran 2015a, b, c, 2016a, b, 2018; Roy et al. 2021; Diukendjieva et al. 2019), but no sufficient physical explanation could be derived from the models’ predictions. A list of QSAR models recently reported in literature for predicting PAMPA permeability, along with information on the statistical methods, the descriptors and data used is presented in Table 1.

**Table 1:** QSAR models for predicting PAMPA permeability

References	Models	Data	Descriptors	Performance	Availability
Chi (2019)	PLS^**‡**^@@HSVR^**‡**^	190 molecules from Oja and Maran (2015a, b, c, 2016a, b, 2018)	MW*, logP*, logD*, PSA*, FPSA*, μ*	*R*^2^ = 0.79 (best performing model HSVM-R, test set)	Data: available Code: not available
Sun (2017)	SVM-R^**‡**^ SVM-C^**‡**^	5.435 molecules (from NCATS database)	Atom-type descriptors built from a universal, generic molecular descriptor system not readily accessible	*R*^2^ = 0.9, AUC = 0.88–0.90	Data and code: not available
Oja (2015a, b, \c, 2016a, \b, 2018)	MLR^**‡**^	Databases of acidic, basic, neutral and amphoteric compounds at different pH values	Hydrophobic/hydrophilic properties at different levels of complexity and hydrogen bonding-related surface areas	*R*^2^ > 0.8 (acidic compounds), *R*^2^ > 0.7 (basic compounds), *R*^2^ = 0.95 (neutral compounds)	Data and code: available
Roy (2021)	GBM^**‡**^ XGBM^**‡**^ GLM^**‡**^, SVM-C^**‡**^, kNN^**‡**^	Molecules from previously published datasets	Excess chemical potentials of drugs/solute molecules in specific solvents using the 3D-RISM-KH molecular solvation theory and 2D molecular descriptors calculated with the publicly available PaDEL software	*R*^2^ = 0.64 (best XGB model, test set), Accuracy = 0.93 (best SVM model)	Data: available, Code: not available
Diukendjieva (2019)	MLR^**‡**^	269 drugs and drug-like compounds	Molecular descriptors calculated with publicly available software	*R*^2^ = 0.74	Data: available, Code: not available

*MW: molecular weight, logP: octanol/water partition coefficient, logD: octanol/water distribution coefficient, PSA: polar surface area, FPSA: fractional polar surface area, μ: dipole moment

^‡^PLS: partial least squares, HSVR: hierarchical support vector regression, SVM-R: support vector machine regression, SVM-C: support vector machine classification, MLR: multiple linear regression, GBM: gradient boosting machine, XGBM: extreme gradient boosting machine, GLM: generalized linear models, kNN: k-nearest neighbours

To bring new insight into the above-mentioned problem, we used the dataset of 190 molecules curated by Chi et al. (2019) to model the PAMPA permeability of molecules. We developed a QSAR approach using a set of 61 selected descriptors that, apart from effectively mapping chemical space, allow for structural interpretation of the molecules. As will be analytically discussed in Sect. 4, the set contains Lipinski-like physicochemical features (Lipinski 2000; Lipinski et al. 2001) as well as BCUT structural descriptors (Stanton 1999) from which chemical structures can be uniquely deduced (Masek et al. 2008) and visualized (Guha and Willighagen 2012), giving clear insight into the influence of different groups on the permeation ability of molecules and vice versa, i.e. how permeability will be affected by structural changes. Drug discovery decisions can be made, as for example a targeted modification of a chemical structure or the selection of a chemical series with promising permeation profile for further refinement.

Using the selected descriptors, we subsequently employed artificial neural network (ANN) algorithms (Günther et al. 2010) to create a highly accurate “stacked regression” ensemble QSAR model (Wolpert 1992; Breiman 1996) to predict the permeation ability of molecules. Well-trained ANN models show increased accuracy and precision and are routinely used to solve complex problems (Irshad et al. 2020; Kaur et al. 2020; Sarker et al. 2020; Alloqmani et al. 2021; Jaber Alsolami et al. 2021). Our ensemble combined two neural network base models generated using a model development dataset split explicitly into a training and a test set. The models were fitted on the training set—using 20-fold cross-validation with three repeats—and validated on the test set to have an early estimation of their predictive performance on new data. The optimization of hidden layers (number of layers and neurons) of the models was based on the values of R-square and root mean-square error (RMSE) (Alexander et al. 2015; Kvålseth 1985) metrics indicating the predictive ability of the models on both datasets. The predictions of the models on the training set were subsequently combined using an ANN algorithm to create a stacked ensemble. The ability of the model to generalize well, i.e. to make accurate predictions on unseen data was further evaluated using independent external validation datasets (Irshad et al. 2020; Ho et al. 2020). Details on model generation, characteristics and performance metrics are provided in Sect. 3 of the manuscript. Our AΝΝ ensemble outperformed the HSVR model reported by Chi et al. (2019), with the Pearson correlation between observed and predicted *logPe* values for the 190 molecules being 0.97 and 0.93, respectively.

On the whole, the contribution of our approach can be summarized as follows:

- we identified a set of 61 theoretically calculated descriptors with high relevance in explaining the permeability of molecules, from which chemical structures can be uniquely deduced;
- we created an ensemble ANN regression model that can predict with high accuracy the PAMPA permeability of compounds of interest in a very short time (5 min to train the models using a system with CPU @ 2.69 GHz and RAM 12 GB).

The ANN ensemble was further used to predict the PAMPA permeability of molecules contributed by medicinal chemists to the COVID Moonshot project and downloaded from the PostEra site (PostEra 2022) (Supporting Information1, sheets S1.6 & S1.7 respectively). Our goal in doing so was to join and strengthen the ongoing efforts towards the development from scratch of new, orally administered, target-specific drugs with optimized absorption profile for COVID-19 treatment.

Briefly, this manuscript has been structured as follows: in Sect. 2, the experimental details of the study are analytically described and a diagram summarizing the workflow is provided; in Sect. 3, the results of the analysis and the creation of the QSAR models are presented; in Sect. 4, the contribution of the present study in predicting permeation ability of molecules is extensively discussed; and Sect. 6 contains information on the working environment and the availability of data and code.

## Experimental

Special consideration was given to the consistency, quality and completeness of the data; hence, a homogenous, publicly available dataset (Chi et al. 2019) (Supporting Information1, sheet S1.1) with recorded PAMPA permeability for 190 molecules measured under the same experimental protocol was used to build the models. This is important, since permeability measurements heavily depend on the applied experimental protocol (Chi et al. 2019; Dahlgren and Lennernäs 2019; Avdeef et al. 2004). Also, as different types of measurements result in different permeability coefficients (Chi et al. 2019; Dahlgren and Lennernäs 2019), we note that in the present work we have modelled the *effective permeability coefficient* (*logPe*), analytically described below.

### Description of the PAMPA method

The PAMPA method measures the permeability via passive diffusion, based on an artificial non-cell lipid membrane without pores, active transport systems or metabolizing enzymes (Fortuna et al. 2007). Used therefore as a HTS method, PAMPA is very successful in establishing the structure–activity relationships (SARs) and hit-to-lead optimization (Fortuna et al. 2007). The PAMPA system is a ‘sandwich’ consisting of two 96-well plates and includes three compartments. Substances move from a donor compartment, through a lipid-infused artificial membrane into an acceptor compartment (Kansy et al. 1998; Chi et al. 2019). The donor, membrane and acceptor compartments emulate the gastrointestinal tract, the intestinal epithelium and the blood circulation, respectively. To date, PAMPA models have been developed that exhibit a high degree of correlation with permeation across a variety of barriers, including the gastrointestinal tract (Avdeef et al. 2004), Caco-2 cultures (Bermejo et al. 2004; Avdeef et al. 2005), blood–brain barrier (Dagenais et al. 2009) and skin (Sinkó et al. 2012). The simplicity and stability of the PAMPA system allow for variability in the experimental settings, e.g. changing the pH values in the donor compartment offers the possibility to measure permeability under different physiological conditions in the intestinal pathway (Berben et al. 2018; Kansy et al. 1998). PAMPA measurements are shown to compare well with human intestinal absorption, except for some problematic cases concerning compounds with limited solubility or specific drug classes and compounds absorbed by active transport (Fortuna et al. 2007).

### Permeability measurements and experimental setup

The influence of pH on the absorption through the intestine of drug-like molecules has been previously reported (Oja and Maran 2015a, b, c, 2016a, b, 2018). Indeed, the intestinal environment may present a variation in terms of pH values that possibly affects the absorption properties of substances (Oja and Maran 2018; Avdeef 2001). In keeping with this, the PAMPA assay has been used to measure the pH-dependent permeability profiles of various compounds (Oja and Maran 2015a, b, c, 2016a, b, 2018).

The present QSAR study is based on the *effective membrane permeability* (Chi et al. 2019; Dahlgren and Lennernäs 2019) measurements initially performed on a series of acidic, basic, neutral and amphoteric compounds at pH 7.4 by Oja and Maran (2015a, b, c, 2016a, b, 2018) and subsequently curated by Chi et al. (2019) in a dataset of 190 selected molecules (Supporting Information S1.1).

The *effective membrane permeability coefficient* (*logPe*) was calculated according to the equation (Oja and Maran 2016a):

$$log \left(Pe\left(\frac{cm}{s}\right)\right)=log \left( -\frac{2.303. {V}_{D}}{A.(t-{\tau }_{ss).{\varepsilon }_{a}}} . \left(\frac{1}{1+{r}_{v}}\right). {log}_{10}\left[1-\left(\frac{1+{r}_{v}^{-1}}{1-{R}_{M}} \right) . \frac{{C}_{A }\left(t\right)}{{C}_{D }\left(0\right)} \right]\right),$$

where ***V***_***D***_ is the volume of the solution in the donor side, ***A*** is the membrane area, ***t*** is the time point of the experiment, ***t***_***ss***_ is the lag time, ***e***_***α***_ is the apparent membrane porosity***, r***_***v***_ is the ratio of volumes of the donor and acceptor sides (*r*_*v*_ = *V*_*D*_*/V*_*A*_), ***C***_***D***_***(0)*** is the initial compound concentration in the donor side, ***C***_***A***_**(*t*)** is the concentration in the acceptor side at time ***t***and ***R***_***M***_ is the membrane retention ratio:

$$R_{M}=1-( \frac{{C}_{D }\left(t\right)}{{C}_{D}\left(0\right)}- \frac{{V}_{A }{C}_{A}(t)}{{V}_{D}{C}_{D}(0)}).$$

As the cutoff value for the membrane permeability depends on the experimental system, we note here that, for the specific experimental setup and for pH 7.4, a rough approximation may be employed (Chi et al. 2019) according to which *logPe* values ≥ -6.2 correspond to compounds with higher permeability, whereas *logPe* values < -6.2 would indicate lower permeability in general.

### Partitioning of the data for model development and validation: calculation of molecular descriptors and model performance statistics

#### Train, test and external validation datasets (supporting information 1, sheet S1.1)

**Fig. 1:**
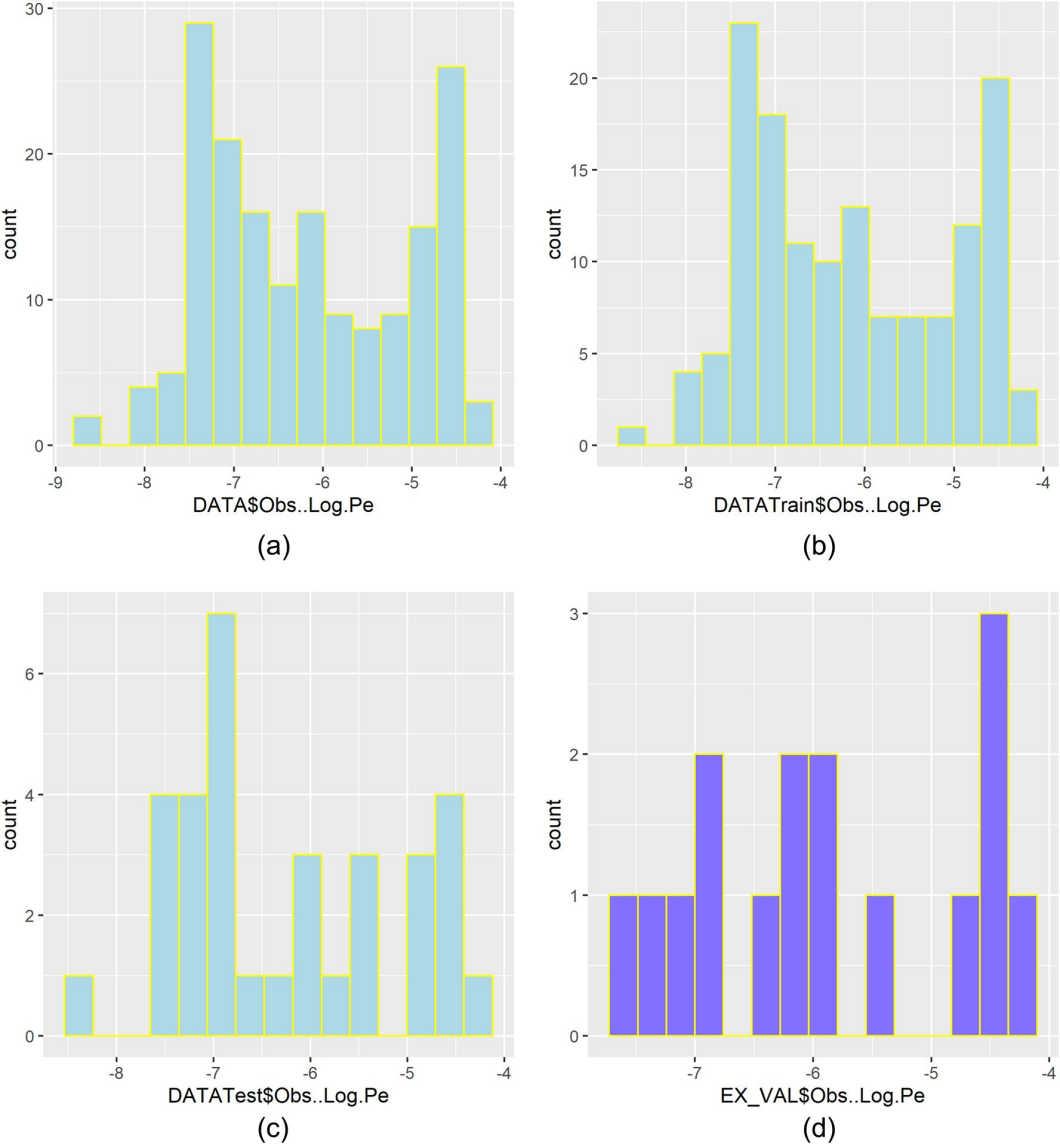
Partition of the data: distribution of the output variable *(logPe*) in the whole dataset as well as in the train, test and external validation subsets

A visualization of the data split for the *logPe* modelling is presented in Fig. 1. The individual subsets were saved as CSV files for reading into the R modelling workflows and these CSV files are provided in the code archive available on Zenodo (Gousiadou 2021), along with a README file explaining their contents and guidance on how to reproduce results via running the available code files.

##### Model development data: train and test subsets

For model development and initial evaluation, a dataset of 174 molecules randomly selected out of the set of 190 compounds was further randomly split into explicit train (80%, 141) and test (20%, 33) subsets. The train set was used to fine-tune the algorithm parameters and fit the models, while the test set provided an early estimate of their predictive performance (Table 2).

**Table 2:** Modelling the effective membrane permeability (logPe) of compounds (190)

A. Creation of models and evaluation of models’ performance on the train set (141), ( 20-fold cross-validation with three repeats)					
Models	^‡^R^**2**^_CV_	R^2^_CV_	RMSE_CV_	Resubstitution	Model layers and nodes
*NN1*	0.58	0.35	0.19	Pearson correlation = 0.99 rmse = 0.012 Rsquare = 0.99 R2 = 0.99	Hidden (layer1 = 20, layer2 = 15, layer3 = 5)
*NN2*	0.56	0.17	0.20	Pearson correlation = 0.99 rmse = 0.008 Rsquare = 0.99 R2 = 0.99	Hidden (layer1 = 30, layer2 = 20, layer3 = 10)

B. Creation of stacked model					
Creation of the stacked model RFEnsembleX by combining the predictions of the models on the train set (141) with the neuralnet algorithm (20-fold cross-validation with three repeats)					
Stacked model (neuralnet)	^***‡***^***Rcv***^***2***^	***Rcv***^***2***^	***RMSE***_***CV***_	***Resubstitution***	Model layers and nodes
***EnsembleNN *** ***(NN1* + *NN2)***	0.99	0.99	0.012	Pearson correlation = 0.99 rmse = 0.013 Rsquare = 0.99 R2 = 0.99	Hidden (layer1 = 2, layer2 = 1, layer3 = 0)

C. Evaluation of models’ performance on the test set (33)					
***Models***	^***‡***^***R***^***2***^	***R***^***2***^	***RMSE***	***Pearson correlation***	
*NN1*	0.82	0.80	0.11	0.90	
*NN2*	0.65	0.61	0.16	0.80	
***EnsembleNN***	0.79	0.78	0.12	0.89	

D. *Evaluation of models’ performance on the external validation set (16)*					
Models	^‡^***R***^***2***^	R^***2***^	***RMSE***	Pearson correlation	
*NN1*	0.75	0.69	0.14	0.86	
*NN2*	0.74	0.71	0.14	0.86	
***EnsembleNN***	0.79	0.76	0.12	0.89	

##### External validation set

For the external validation of the final models, we used the following sets of data (Supporting Information 1, S1.1): **a.** 16 molecules initially partitioned from the dataset of 190 compounds, which were set aside to create an independent external validation set; **b.** a set containing the chemical structures of two anti-COVID-19 drugs, namely Paxlovid (Owen et al. 2021) and Remdesivir (Jang et al. 2021) with reported permeability (not PAMPA). The SMILES strings of the drugs were downloaded from the PubChem database (Sayers et al. 2022).

#### Calculation of molecular descriptors

A single 3D conformation was created from SMILES for each structure using the publicly available Bioclipse software (Spjuth et al. 2007, 2009). An SDF file containing the 3D coordinates of the molecules was imported in *R*, and the *rcdk* package (Guha 2007) was used to automatically calculate a number of descriptor variables. The CDK descriptors (Java Library for Chemoinformatics) are divided broadly into three main groups, that is, atomic, bond and molecular and belong to the specific categories “topological”, “geometrical”, “hybrid”, “constitutional”, and “electronic”. The calculation resulted in 286 descriptors for each molecule. Non-informative descriptors were removed, that is, all variables with zero variance (zero values for all molecules). This process reduced the number of descriptors to 232.

#### Model performance statistics

For the comparison and evaluation of the predictive performance of models, we primarily employed the Pearson’s correlation coefficient, the coefficient of determination (*R*^2^, Eqs. 1 and 2) and the "root mean-square error" (RMSE, Eq. 3) metrics (Alexander et al. 2015; Kvålseth 1985). The best models were those with the smaller RMSE and greater *R*^2^ values. Whilst different *R*^2^ (“Rsquared”) and related statistics may be reported in the literature (Alexander et al. 2015; Kvålseth 1985; Roy et al. 2009), here we employed Eqs. (1), (2) and (3) recommended as generally suited for QSAR studies (Alexander et al. 2015; Kvålseth 1985). Assuming that the difference between the mean experimental and predicted values is zero, “R-squared” can be interpreted as the proportion of the variability in the response captured by each model (Alexander et al. 2015; Kvålseth 1985). However, under certain circumstances, e.g. due to the average prediction being significantly shifted from the average experimental value or due to outliers, *R*^2^ (Eq. 1) can be negative.

We note that, where statistics are reported with the subscript “cv” (*R*^2^_CV,_^‡^*R*^2^_CV,_ RMSE_CV_), this means that the model built on a cross-validation training subset was applied to the corresponding validation fold, with the performance statistic being averaged across all folds and repeats of cross-validation. (Supporting Information1, sheet S1.4). The coefficients of determination reported as *R*^2^ and *R*^2^_CV_ were calculated using Eq. (1), whilst the coefficients of determination ^‡^*R*^2^ and ^‡^*R*^2^_CV_ were calculated using Eq. (2). For the coefficients of determination depicted as *R*^2^ and ^‡^*R*^2^ (without the “cv” indication), the corresponding calculations were made by applying the models to data other than those used to train them, i.e. the test and external validation sets. Where correlation statistics are referred to as “resubstitution” estimates, this means that the model trained on the training set was applied to that training set (Hawkins 2004). These are not estimates of predictive performance, but may provide insight into the degree of overfitting when compared to the corresponding statistics on truly independent data.

1 $$\mathrm{R}2 =1-\frac{\sum {\left(y-\hat{y}\right)}^{2}}{\sum {\left(y-\bar{y}\right)}^{2}},$$

2 $$\ddagger \mathrm{R}2 = {\left(\frac{cov\left(y,\hat{y}\right)}{\sqrt{var\left(y\right). var\left(\hat{y}\right)}}\right)}^{2},$$

3 $$\mathrm{RMSE }=\sqrt{\frac{\sum_{i=1}^{N}{\left({y}_{i}-{\hat{y}}_{i}\right)}^{2}}{N}},$$

where *y* and *ŷ* are the observed and predicted values, respectively, and $\bar{y}$ is the mean of the observed values.

#### Workflow for model development and validation

**Fig. 2:**
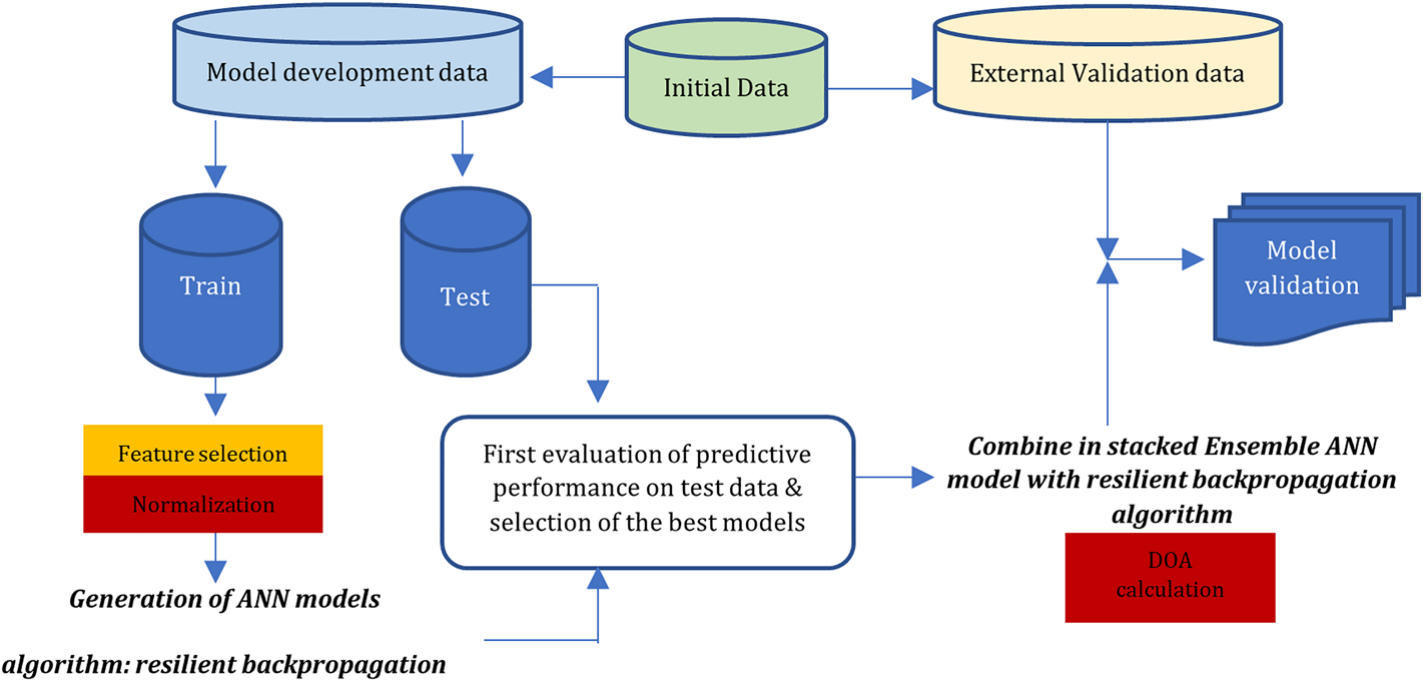
Diagram depicting the various steps included in the present computational analysis, i.e. data separation, pre-processing and feature selection, development and validation of the models

The workflow followed in this study is summarized in Fig. 2. The different stages of the analysis are clearly shown, i.e. data separation, data pre-processing and feature selection as well as the development and validation of the models.

## Results

### Data pre-processing and feature selection

**Fig. 3:**
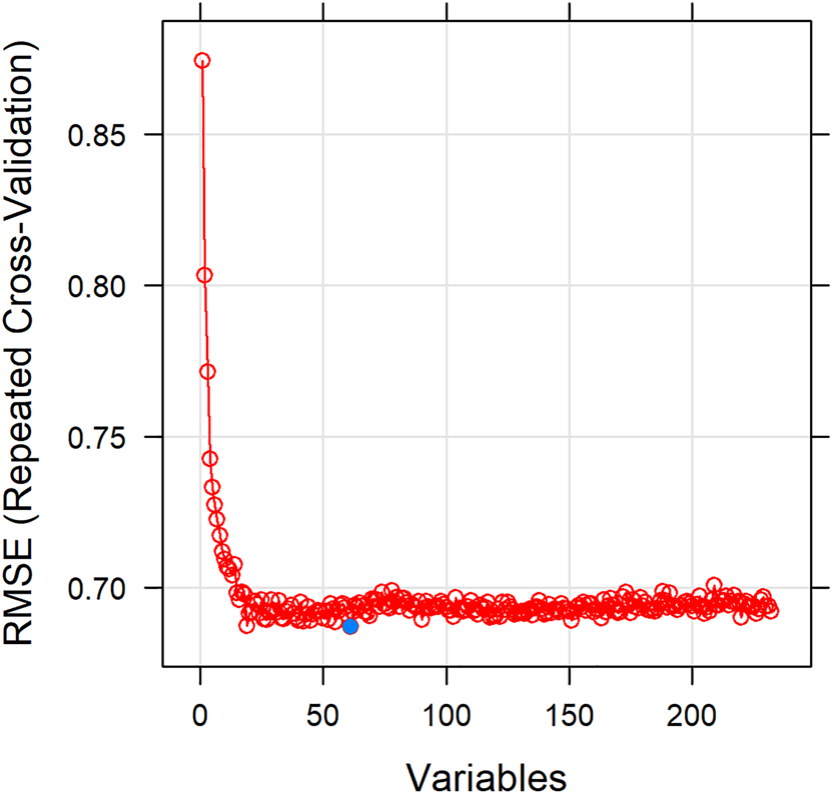
Selection of descriptors. Feature selection with random forest (recursive feature elimination) for the *effective permeability* (*logPe*) modelling, using the 141 molecules included in the train set. The best performance based on the root mean-square error (RMSEcv) (Kaur et al. 2020) corresponded to a subset of 61 descriptor variables selected as most significant in predicting the *logPe* values

An initial exploratory analysis of the dataset (190 molecules, 232 descriptors) revealed a high correlation (> 0.80) between the 127 descriptors. As it is always desirable to have a reduced set of uncorrelated, nonredundant, and informative descriptors that allow for interpretable prediction models, we reduced data dimensionality using feature elimination methods. Feature selection was performed using the training set of 141 molecules with 232 descriptors and the corresponding *logPe* values. The method selected for the feature elimination was based on a wrapper approach (John et al. 1994). Wrapper methods are search algorithms that treat the predictors as inputs and utilize model performance as the criterion to be optimized (Ambroise and McLachlan 2002). Using the *caret* package (version 6.0–84) in R, we performed a simple backwards selection of descriptors (Recursive Feature Elimination, RFE) with random forest (*randomForest* package—version 4.6–14) (Svetnik et al. 2003). Random forest has a built-in feature selection (Svetnik et al. 2004) as well as variable importance estimation utilized for the RFE approach (Kuhn 2019; Kotu and Deshpande 2019). We used the version of the algorithm that incorporates resampling (*rfe*) (Kuhn 2019) and applied an outer resampling method of 20-fold cross-validation with three repeats to reduce the risk of overfitting of the model to the descriptors and to get performance estimates that incorporate the variation due to feature selection. By employing the resampling method, we improved the generalization performance of the model and obtained a more probabilistic assessment of descriptor importance than a ranking based on a single fixed data set. The best performance was based on the Root Mean-Square Error (RMSE_CV_) (Alexander et al. 2015) and corresponded to a subset of 61 descriptor variables—ranked according to their significance in predicting the *logPe* values (Fig. 3), (Supporting Information1, sheet S1.5)—which we further used to build our models.

### QSAR models created using the selected 61 descriptors

The data in the train set were subsequently pre-processed, i.e. normalized in the range 0–1. The same pre-process parameters were applied for normalization of the test (33 molecules) and external validation (16 molecules) datasets. Next, we used the training data and employed a sophisticated ensemble modelling approach known as “stacked regression” (Breiman 1996). Ensemble approaches combine the predictions of multiple learning algorithms for obtaining improved predictive performance, which could not otherwise be obtained from any of the constituent learners alone. Although an ensemble may have multiple base models within the model, it acts and performs as a single model (Kotu and Deshpande 2019). The advantage of such a “metalearner” is that the generalization error of the prediction is minimized by deducing the biases of the base models with respect to a provided learning set. This deduction proceeds by generalizing in a second space—whose inputs are the predictions of the base learners on a given dataset and whose output is the actual outcome—and trying to make predictions on new, unseen data (Wolpert 1992).

For the present regression analysis, we used the resilient backpropagation method (Günther et al. 2010; Riedmiller and Braun 1993) to generate feedforward neural network models (Hornik et al. 1989) to be combined in an ensemble. This method is considered one of the fastest for regression analyses and does not require predefining of the overall learning rate. We employed the resilient backpropagation algorithm with weight backtracking (*rpart* +) (Svetnik et al. 2003), available in the *neuralnet* package (version 1.44.2) (Günther et al. 2010), and trained multi-layer perceptrons (MLPs) that predicted permeability by calculating the following function:

$$ \begin{aligned} o\left(x\right)& =f\left({w}_{0}+\sum_{j=1}^{j}{w}_{j}.f\left({w}_{oj}+\sum_{i=1}^{n}{w}_{ij}{x}_{i}\right)\right)\\&=f\left({w}_{o }+\sum_{j=1}^{j}{w}_{j}.f({w}_{oj}+{{\varvec{w}}}_{j}^{ T}{\varvec{x}})\right),\end{aligned} $$

where w_0_ denotes the intercept of the output neuron, w_*oj*_ the intercept of the *jth* hidden neuron, *w*_*j*_ the synaptic weight that corresponds to the synapse starting at the *jth*, hidden neuron and leading to the output neuron, ***w*** = (*w*_*1j*_*,…, w*_*nj*_) the vector of all synaptic weights corresponding to the synapses leading to the *jth* hidden neuron, and ***x*** the vector of all covariates (*x*_*1*_*,…, x*_*n*_). More specifically, a number of neurons are organized in consequent layers connected by synapses, and the output of every neuron in one layer is the input to a neuron in the next layer. All the covariates (descriptors) are arranged in separate neurons to form the input layer, while the output layer consists of the response variable. The intermediate layers are referred to as hidden layers. A weight indicating the effect of the corresponding neuron is attached to each one of the synapses (Günther et al. 2010). These weights are the parameters of the backpropagation ANN models and during the training process are modified by the algorithm to minimize the error function that measures the difference between the observed (*o*) and predicted (*y*) output values:

$$E=\frac{1}{2}\sum_{l=1}^{L}\sum_{h=1}^{H}{{(o}_{ith}-{y}_{ith})}^{2},$$

where *l* = 1,…,*L* is the index for the observations and *h* = 1,…,*H* is the index for the output nodes.

The algorithm *rpart* + uses only the sign instead of the magnitude of the partial derivatives to update the weights. Based on this method and using the previously selected 61 descriptors, we trained a series of neural network learners on the train set of 141 molecules to compare their performance. As the selection of the number of hidden units in an ANN is not an exact science, trial and error played a significant role in this process. The algorithm was applied on the training data using different number of hidden layers and neurons and employing a resampling method of 20-fold cross-validation with three repeats. The final choice of hidden units (Table 2) was based on a compromise between the quality of the model (learning well from the data, avoiding overfitting, etc.) and complexity/computational speed. However, it was observed that the use of a relatively small number of covariates (61 descriptors) significantly reduced the complexity of the models, despite increasing the hidden layers and neurons in a number of them. Further fine-tuning regarding the hidden layers was performed based on the resulting root mean-square error and R-squared values (RMSE_CV_ and ^‡^R^2^_CV_)—calculated according to Eqs. (3) and (2), respectively (described in “Model Performance Statistics” section) and presented as the average across all folds and repeats of cross-validation. The models were subsequently used to predict the *logPe* values of the 33 molecules in the test set, which provided a less biased evaluation of the models’ effectiveness in predicting unseen data. Based on the results from both datasets, two ANN based learners with optimized parameters were finally selected to be combined in a stacked ensemble (Table 2B).

**Fig. 4:**
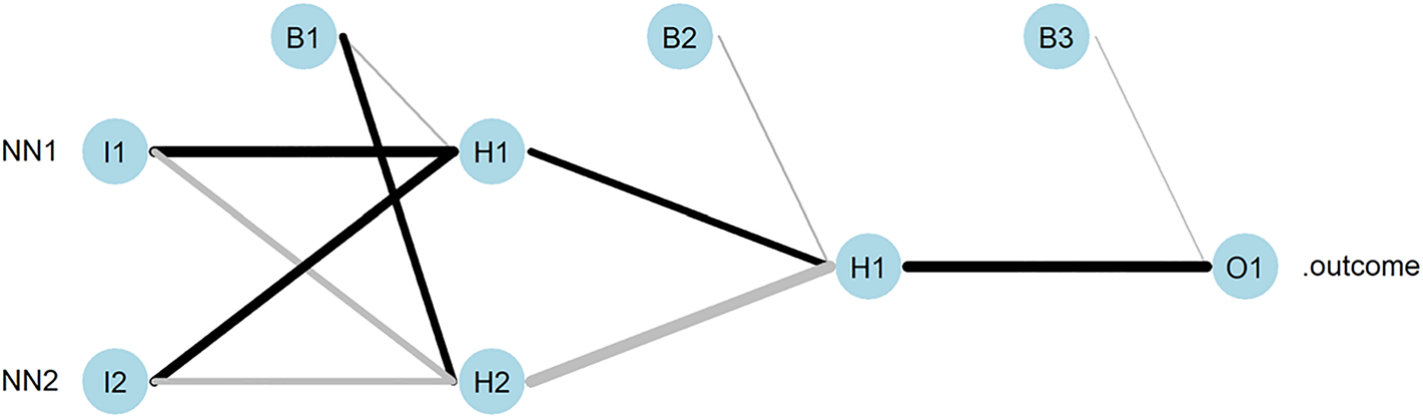
Architecture and complexity of the *EnsembleNN*. As input variables for the ensemble, NN1 and NN2 are used, i.e. the *logPe* values predicted by the neural network base models *NN1* and *NN2*, respectively, for the molecules in the training dataset. The observed *logPe* values of the molecules is the output of the model. The ensemble further consists of two hidden layers and three hidden neurons. The weights are depicted by black (weights with positive sign) and grey (weights with negative sign) lines. The result matrix is presented in Table 3

The architecture and complexity of the *Ensemble NN* model is analytically presented in Table 3 and Fig. 4.

**Table 3:** Result matrix for the EnsembleNN

Error	1.19E-02
reached.threshold	9.76E-03
steps	1.43E + 03
Intercept.to.1layhid1	– 5.09E-01
NN1.to.1layhid1	2.10E + 00
NN2.to.1layhid1	1.86E + 00
Intercept.to.1layhid2	1.58E + 00
NN1.to.1layhid2	– 1.24E + 00
NN2.to.1layhid2	– 1.29E + 00
Intercept.to.2layhid1	– 4.29E-01
1layhid1.to.2layhid1	1.41E + 00
1layhid2.to.2layhid1	– 2.38E + 00
Intercept.to..outcome	– 2.09E-01
2layhid1.to..outcome	2.08E + 00

**Fig. 5:**
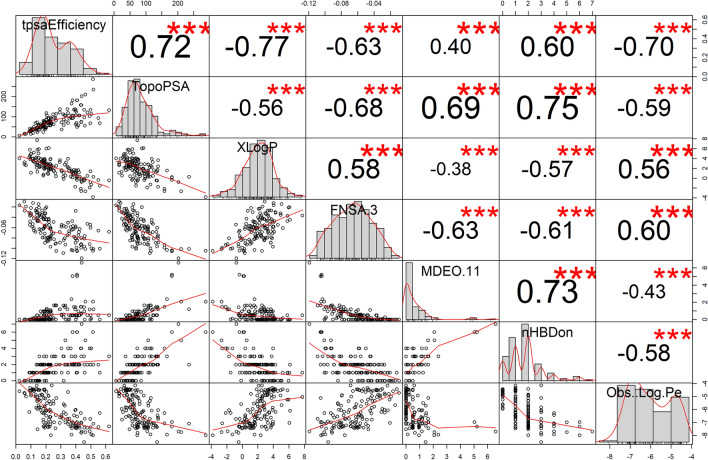
Correlation chart of the top 6 out of 61 most important descriptors, along with the modelled end point ***Observed Log Pe***, for the modelling of membrane permeability (by passive diffusion) of 190 molecules. The distributions of the variables, their correlation to each other and to the output as well as their individual contribution in explaining the variability of the output *Observed Log Pe* is depicted. The Pearson correlation coefficient is reported for each pairwise comparison, with the number of stars assigned increasing with the magnitude of the correlation

An overall evaluation of the variable importance performed by the models *NN1* and *NN2* while being generated, along with a description of the 20 variables selected as most informative by this process, is provided in Supporting Information1, sheet S1.5. In addition, Fig. 5 presents a correlation chart of the top 6 out of the 20 most important descriptors, along with the modelled end point *Observed Log Pe*. The distributions of the variables, their correlation to each other and to the output as well as their individual contribution in explaining the variability of the output are depicted.

**Fig. 6:**
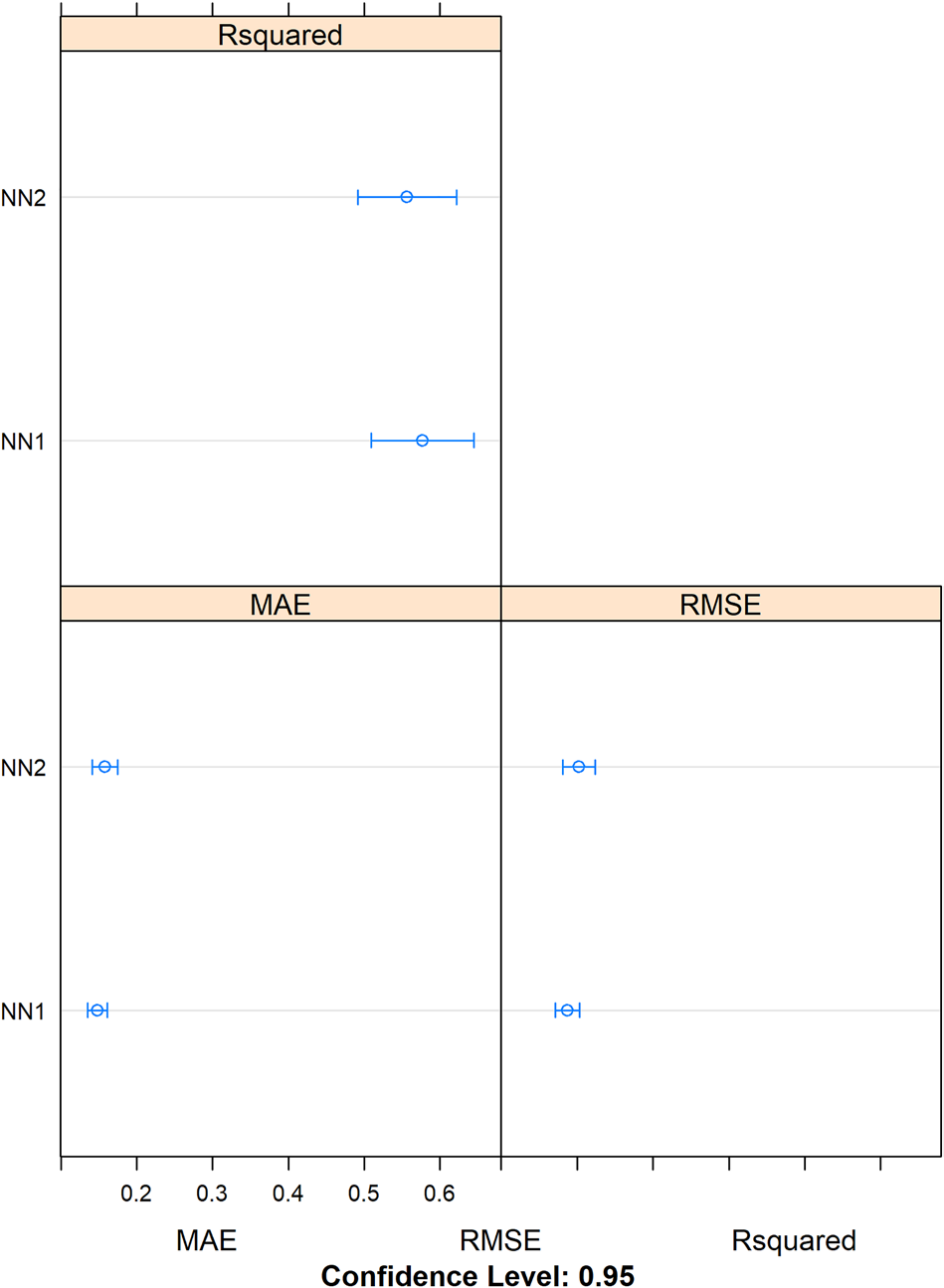
Visual comparison of the modelling results: evaluation metrics *(‡R2*_*CV*_, *RMSE*_*CV*_ and *MAE*_*cv*_) for the prediction performance of the models ***NN1*** and ***NN2*** obtained via cross-validation on the training set (141 molecules) with optimized parameters (Table 2A). The arithmetic mean (circles) and confidence intervals (95%) are plotted for each distribution. Here, “R-squared” refers to *‡R2*_*CV*_, calculated according to Eq. (2) as described in the “Model Performance Statistics” section. The mean absolute error (MAE) (Willmott and Matsuura 2005) evaluation metric, also presented here, is less sensitive to outliers than RMSE_CV_

A visual comparison of the modelling results—based on the evaluation metrics *‡R2*_*CV*_, *RMSE*_*CV*_ and *MAE*_*cv*_ (Willmott and Matsuura 2005)—for the predictive performance of the models *NN1* and *NN2* obtained via cross-validation on the training set (141 molecules) with optimized hyperparameters is depicted in Fig. 5. In addition, in Table 4 and Fig. 6, a pairwise comparison of the cross-validated results for the selected models *NN1* and *NN2* is shown. As can be seen, the correlation between the two models is very low (0.40), which means that each model has captured different aspects of the data and the information they provide has limited redundancy. They are therefore well suited to be combined in an ensemble.

**Table 4:** Pairwise comparison of the cross-validation results for the selected and optimized models *NN1* and *NN2* (Table 1A). The metric used is root mean-squared error (RMSE). The models were not strongly correlated (0.40), indicating that they were informative in different ways and suitable to be combined in an ensemble

*Models*	*NN1*	*NN2*
*NN1*	1.0000000	0.3963362
*NN2*	0.3963362	1.0000000

**Fig. 7:**
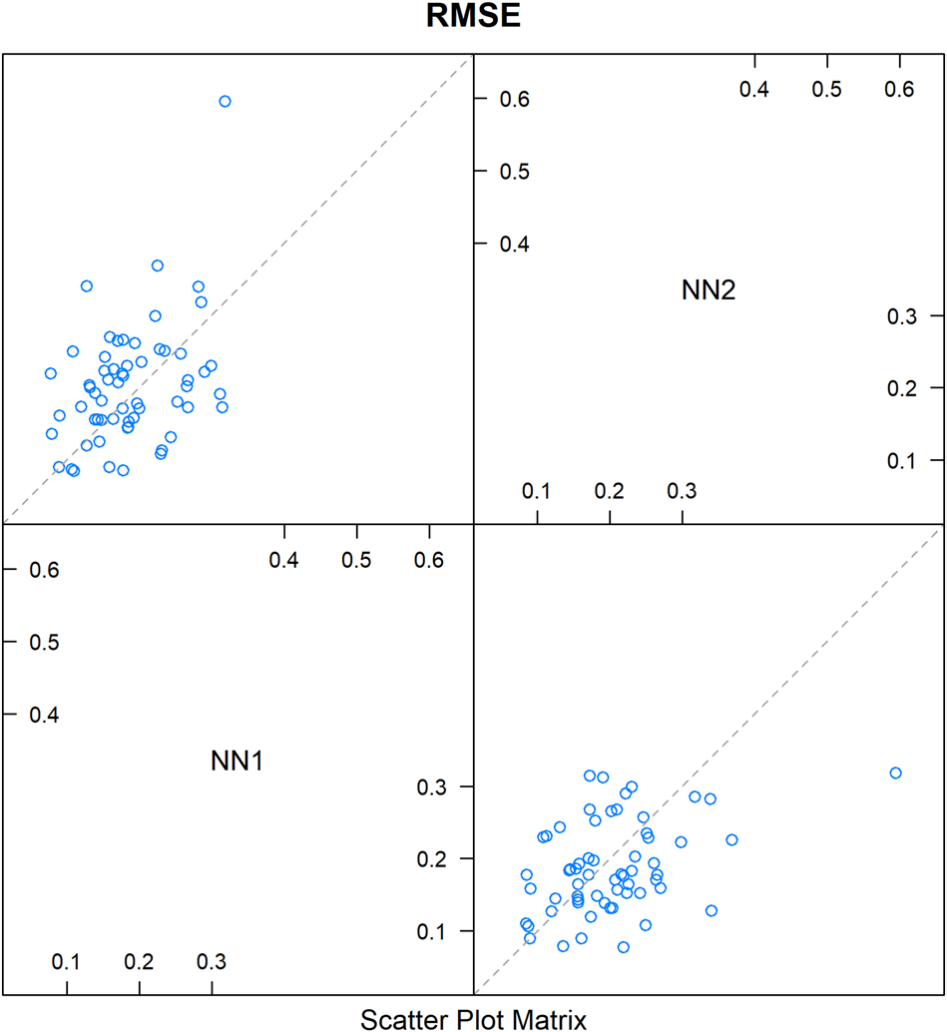
Pairwise comparison of the cross-validation results for the models ***NN1*** and ***NN2*** (Table 4). The scatterplot matrix shows whether the predictions from the models are correlated. The plotted results, for which correlations are examined, are based on the root mean-squared error (RMSE_CV_). If any two models are 100% correlated, they are perfectly aligned around the diagonal. Between ***NN1*** and ***NN2***, the correlation is very low (0.40), meaning that there is limited redundancy in the information given by these models. This proved valuable for the creation of the ensemble model ***EnsembleNN*** (Table 2B)

**Fig. 8:**
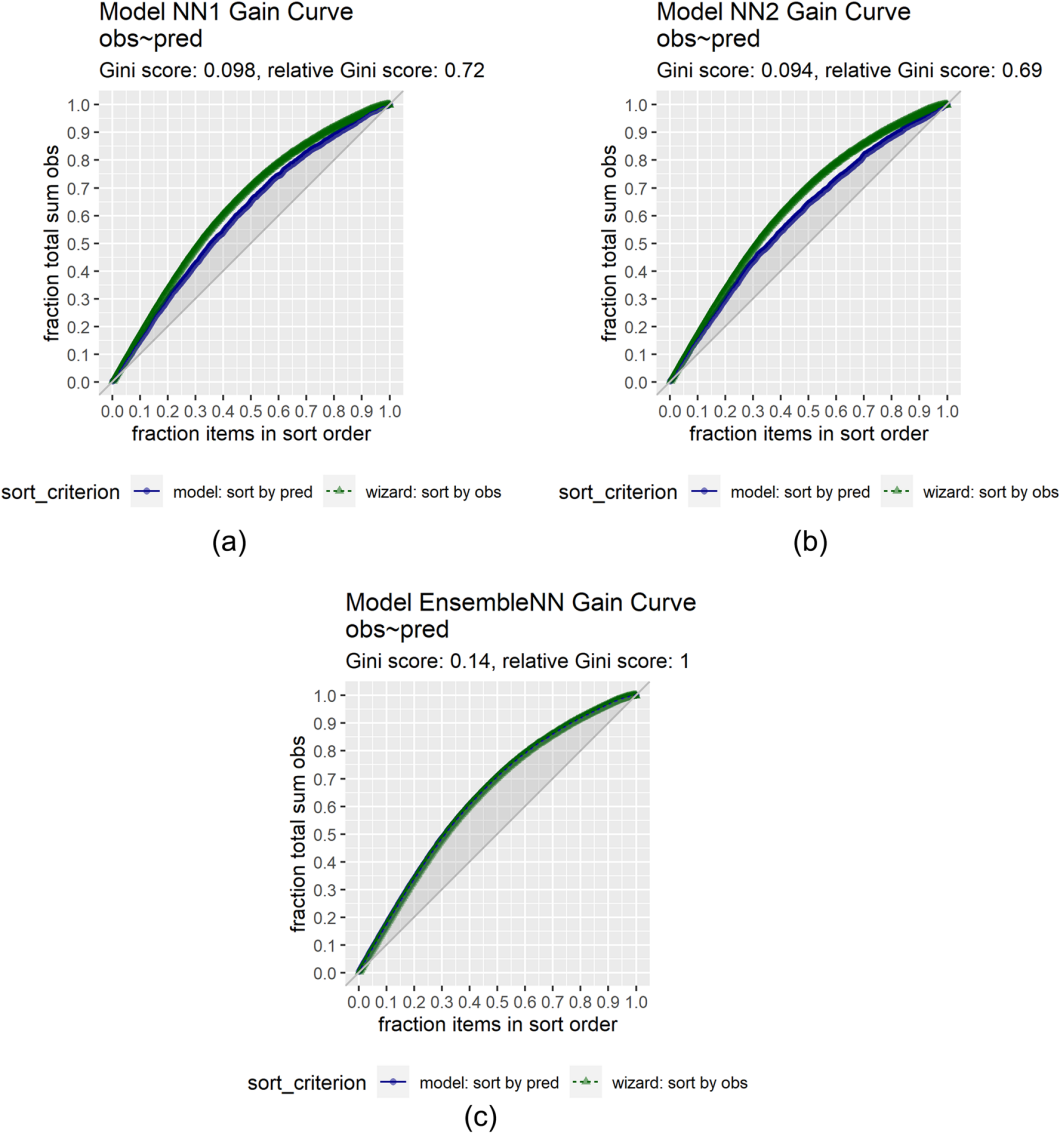
Gain curve plots of the *log Pe* values predicted by the base models ***NN1*** and ***NN2*** and the ensemble model ***EnsembleNN*** against the experimental *logPe* values. The gain curves show whether the models’ predictions are sorted in the same order as the actual *log Pe* values. As sorting is the process of placing elements from a collection in some kind of order, the gain curve plot depicts how well the models sort their predictions compared to the true outcome values. For the evaluation of a model’s performance, the **relative Gini score metric** is used as follows: relative Gini score equals 1 when a model sorts exactly in the same order as the actual outcome, whereas the score is close to zero, or even negative when a model sorts poorly compared to the actual values. The metric therefore can be considered as a measure of how far from “perfect” a model is. The models ***NN1***, ***NN2* and *EnsembleNN*** show relative Gini scores **0.72**, **0.69** and **1**, respectively (Mount and Zumel 2020)

We subsequently trained an ANN stacked ensemble (Table 2B)—employing again the resilient backpropagation algorithm and applying 20-fold cross-validation with three repeats—using as input variables the predictions of the base models on the train set and as output (target) variable the corresponding experimental values of *logPe*. The whole process resulted in the creation of the ensemble model *EnsembleNN* with boosted predictive performance (Fig. 7). In Fig. 8, an illustration of the performance of the base models *NN1* and *NN2* as well as the *EnsembleNN*—obtained via cross-validation on the training set (141 molecules)—is provided by evaluation curves that assess the performance of the models and compare the results with the random pick (baseline) (Mount and Zumel 2020).

### Domain of applicability (DOA) of the ensemble NN model

For estimating the applicability domain (AD) of the ensemble, we employed the standard deviation (SD) method, extensively used in ensemble modelling (Cao et al. 2017; Tetko et al. 2008; Sushko et al. 2010). SD measures model reliability by incorporating information about the model itself and is based on the assumption that if for a given compound the predictions of the models in the ensemble differ significantly, then the ensemble prediction for this compound is likely to be unreliable. For a set of predictions concerning a compound *j* given by a set of *k* trained models in the ensemble, SD is calculated using the following equation:

4 $$SD\left(j\right)=\sqrt{\frac{{\sum }_{i=1}^{k}{{(y}_{i}-\bar{y})}^{2}}{k-1}},$$

where y_*i*_ is the prediction of the *i*th model for compound *j* and ȳ is the mean prediction y_*j*_(*i*) (*i* = 1..*k*).

**Fig. 9:**
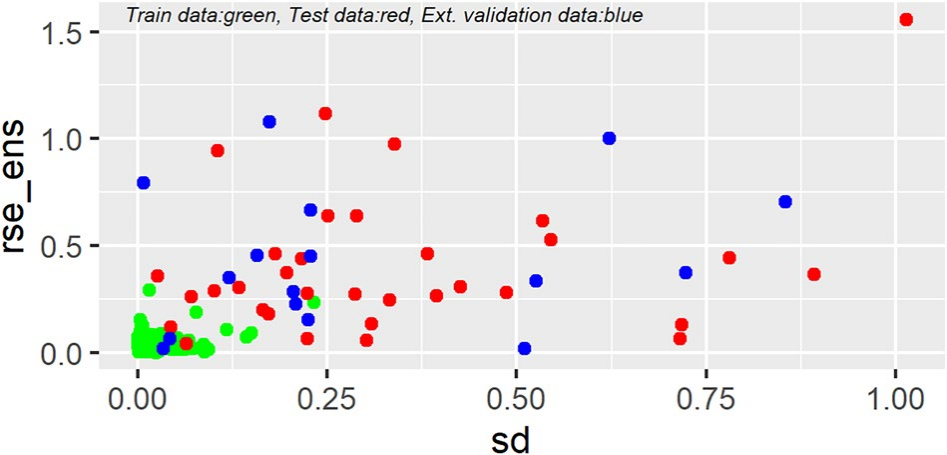
Combined plot depicting the standard deviation (sd) values calculated according to Eq. (4) for the train, test and external validation data versus the root square error (rse_ens) between the respective observed *logPe* values and the predictions made by the *EnsembleNN* model for each one of the molecules. The applicability domain (AD) threshold for the *EnsembleNN* is ~ 3*maxSDTrain (~ 0.69) (Mount and Zumel 2020). For new samples with sd values larger than the threshold, the *logPe* predictions are likely to be inaccurate. Indeed, it is clearly shown that for the molecule with sd > 1 that the difference between the observed and predicted *logPe* values is considerable (rse_ens > 1.5), and had it been a new sample the prediction would rightly not have been considered valid.

The *EnsembleNN* has an AD threshold of approximately three times the maximum SD value of the train data (3*0.23) (Cao et al. 2017), within which the bulk of the ensemble predictions are shown to be reliable (Fig. 9). For new samples with sd values larger than the threshold, the *logPe* predictions are likely to be inaccurate.

**Fig. 10:**
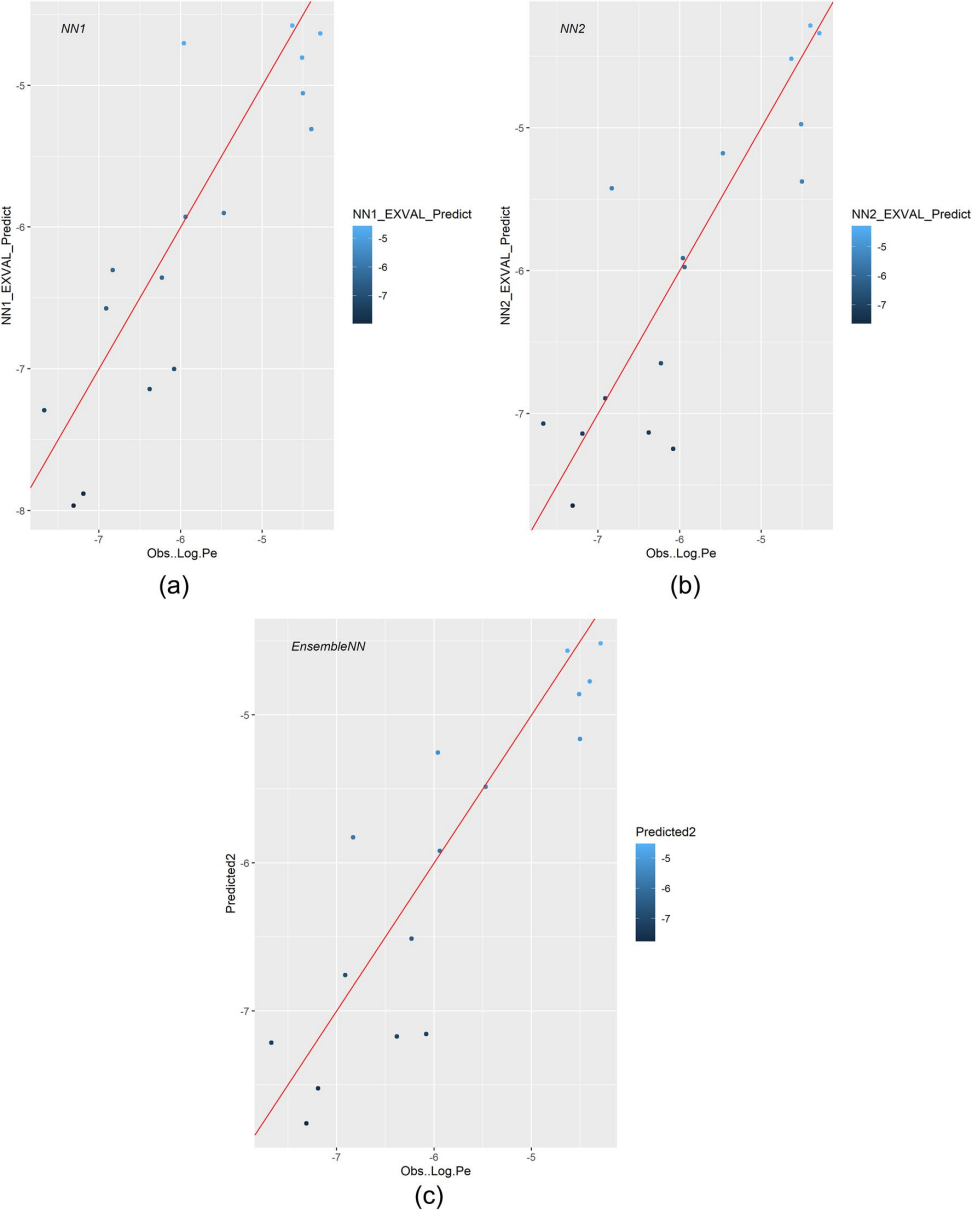
Plot depicting the Pearson correlation (%) of the experimentally observed *logPe* values of the molecules in the **external validation** set versus the values predicted by the base models ***NN1*** (86%) and ***NN2*** (86%) and the stacked regression model ***EnsembleNN*** (89%) (Table 2D)

Subsequently, we evaluated the ability of the *EnsembleNN* to make accurate predictions on the hitherto unseen data of the two external validation sets (normalized with the same parameters used for data pre-processing in the train set). These predictions were completely unbiased, since the external validation sets had not in any way participated previously in the development or selection of the base models (Supporting Information 1, S1.1). On the first external validation set of the 16 molecules the *EnsembleNN* showed enhanced performance, making predictions with 89% correlation to the observed *logPe* values (Table 2D, Fig. 10). The second external validation set consisted of two anti-COVID-19 drugs, namely Paxlovid and Remdesivir for which the permeation ability (not measured with PAMPA) is known (Hung et al. 2022; Schäfer et al. 2022). The two drugs have chemical structures very different from those included in the dataset of 190 molecules: Paxlovid is a new, orally administered, target-specific antiviral drug that has excellent permeation ability and is currently state-of-the-art treatment against COVID-19. Remdesivir, a nucleoside analogue, has been used in the early stages of the pandemic after drug repurposing and due to its low permeability was administered only intravenously. The model made correct predictions within the applicability domain for both molecules: high permeability was predicted for Paxlovid and poor permeability for Remdesivir (predicted *logPe* – 5.29, sd = 0.44 and – 6.72, sd = 0.18, respectively).

Backpropagation depends heavily on the training data (Schäfer et al. 2022). To assert the robustness of the *EnsembleNN*, we have tested model generalization performance as noise rises in the training data. Since in our analysis the chemical structures are represented by theoretically calculated descriptors, noise would only be expected in the output variable, i.e. the experimentally measured *logPe*. We therefore added noise to the *LogPe* values in the training data and repeated the analysis. The results are presented in Supporting Information 1, S1.8.

**Fig. 11:**
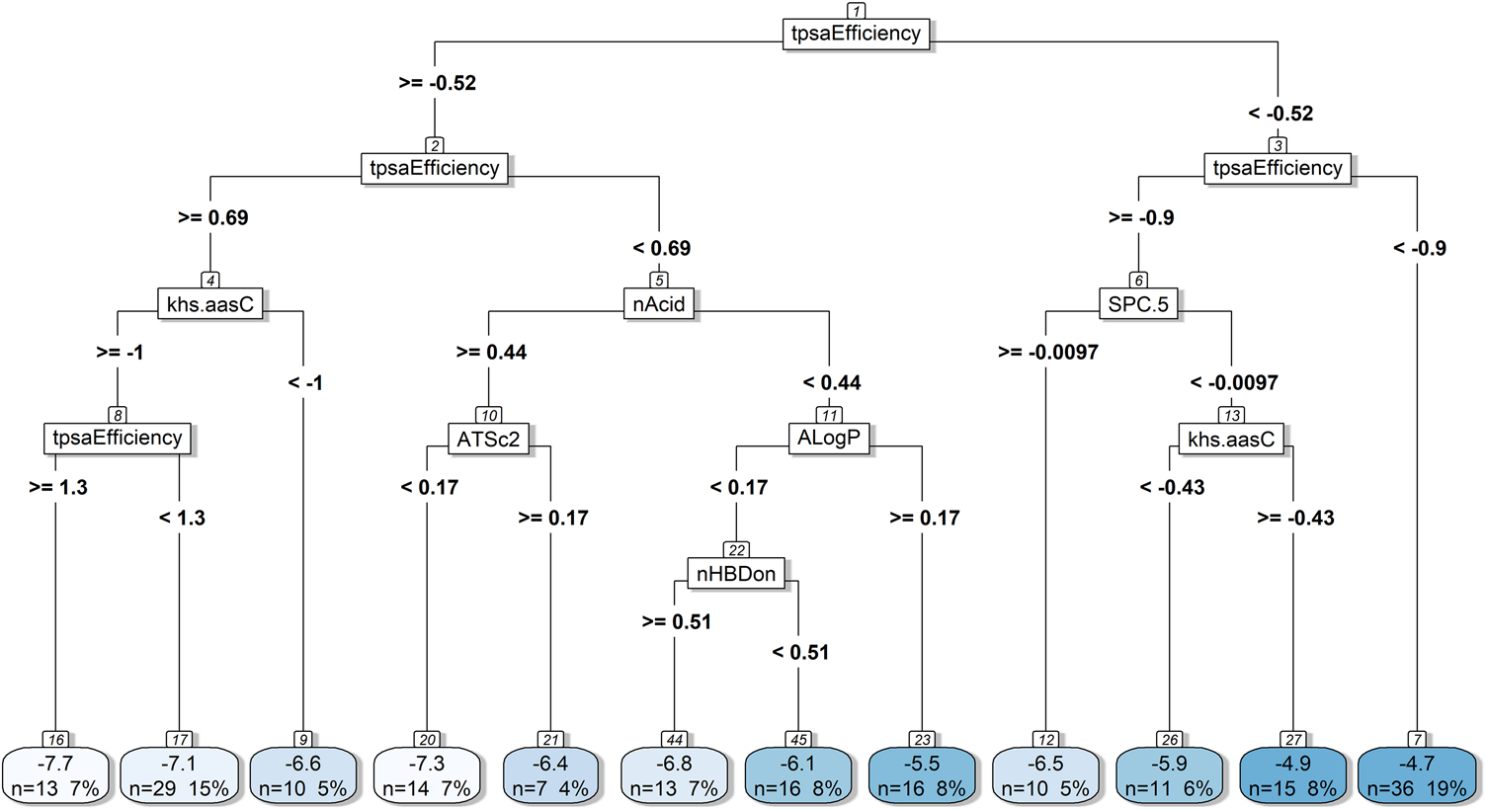
Single decision tree created on the whole dataset (190 molecules) using the 61 descriptors selected by recursive feature elimination (RFE) with random forest. The descriptors’ values are scaled and centred. The decision path clarifies which features are associated with every decision as well as the threshold values of the top descriptors that are responsible for a molecule having high/low *effective permeability* (*logPe*) at pH 7.4. The results are presented in mean values of *logPe*, along with the number and percentage of molecules corresponding to these values. The *logPe* values of the 190 molecules are depicted progressively from white (low permeability) to deep blue (high permeability). According to the rough classification scheme introduced in the section “Permeability Measurements and Experimental Setup” where the cut-off *logPe* value is − 6.2 (Chi et al. 2019), the tree classifies 94 molecules as having “higher permeability” (*logPe* ≥ -− 6.2) and 96 as having “lower permeability” (*logPe* < -− 6.2), whilst 92 and 98 molecules are experimentally shown to have high/low permeability, respectively, according to the PAMPA assay results

Whilst the ANN models can make highly accurate predictions, they provide little explanatory insight into the relative influence of the independent variables in the prediction process (Olden et al. 2004). On these grounds, a more straightforward means of obtaining general insights into the influence of individual descriptors on the modelled *logPe* variable has been employed here. We used the *rpart* (Therneau and Atkinson 2018) algorithm to create a single decision tree on the entire dataset of 190 molecules, using the selected set of 61 descriptors. The decision path (Fig. 11) shows the features—along with their threshold values—associated with every decision. A description of the features is provided in Supporting Information1, S1.5.

### Linear model created using only structural descriptors

To explore the percentage of the *LogPe* variation that a model built on descriptors other than the Lipinski-like properties could explain, we used a subset of 6 descriptors (out of the 61) carrying structural information to build a linear model (Faraway 2005). The set included four structural descriptors from the BCUT chemical space as well as the geometrical FNSA.3 and the MDEO.11 descriptors (Supporting Information 1, sheet S1.5) (Guha 2007). Following the same protocol as previously described (same data partitioning to train, test and external validation datasets, normalization, etc.) we created a linear regression model, and the results are presented in Table 5.

**Table 5:** Modelling the effective membrane permeability (logPe) of compounds (190)

A. Creation of the linear regression model and performance evaluation on the train set (141), ( 20-fold cross-validation with three repeats)				
*Model*	^*‡*^*R*^*2*^_*CV*_	*R*^*2*^_*CV*_	*RMSE*_*CV*_	*Resubstitution*
*LM*	0.43	0.31	0.20	Pearson correlation = 0.64 rmse = 0.20 Rsquare = 0.41 R2 = 0.41

*B. Evaluation of Model’s Performance on the Test Set (33)*				
*Model*	^*‡*^*R*^*2*^	*R*^*2*^	*RMSE*	*Pearson correlation*
*LM*	0.53	0.47	0.18	0.73

C.* Evaluation of model’s performance on the external validation set (16)*				
*Model*	^*‡*^*R*^*2*^	*R*^*2*^	*RMSE*	*Pearson correlation*
*LM*	0.39	0.38	0.20	0.64

As can be seen, approximately 41% of the *LogPe* variation is captured by the six descriptors, with the Pearson correlation between the experimental and the predicted *LogPe* values being 64% for the train and external validation sets and 73% for the test set.

### Implementation of the *EnsembleNN* model

Following development and validation, we used the ensemble model *EnsembleNN* to predict the *logPe* at pH 7.4 of 4520 molecules contributed by medicinal chemists to the COVID Moonshot Project and downloaded from the PostEra site (Kansy et al. 1998) on May 1st, 2020. Our engagement with this data emerged as an activity within the European Union’s Horizon 2020 project NanoCommons Translational Access (TA) (NanoCommons Translational Access (TA) xxxx) and was initiated by Tim Dudgeon from the software company Informatics Matters Ltd. (Informatics Matters Ltd xxxx), who created a repository project board on GitHub (Dudgeon xxxx) dealing with the ADME (Absorption, Distribution, Metabolism and Excretion) analysis of the molecules included in the above-mentioned dataset, for which activity data were not available. As a follow-up, on February 2nd, 2021, we also downloaded from the PostEra site 1561 molecules for which biological activity is available and made predictions on their *logPe* values.

The data were provided as SMILES strings of the molecules, from which a single 3D conformation was created for each structure with the publicly available Bioclipse software (Spjuth et al. 2007, 2009) and the previously selected 61 descriptors were calculated using the *rcdk* package in R. Pre-processing of the data (range from 0–1) was performed with the same parameters used for the development and external validation datasets. Predictions on the permeability of the molecules were performed with the ensemble model *EnsembleNN*, and the results together with data on the molecules´ activity (when available) are presented in Supporting Information S1, sheets S1.6 and S1.7.

Both Rapidfire and fluorescence assays were used to measure the bioactivity of the molecules. The reported IC_50_ values (uM) represent the average over multiple dose–response runs of the molecules in each assay (https:, , github.com, postera-ai, COVID_moonshot_submissions. xxxx; Lu et al. 2016).

The structures of selected molecules (47 out of 1561) exhibiting inhibitory activity against M^pro^ with IC_50_ < 100 uM in both assays, along with their predicted *logPe*, are depicted in Supporting Information 2.

## Discussion

The fact that a drug’s transport via passive diffusion is strongly connected to certain physicochemical properties has been previously shown by Lipinski (Lipinski 2000; Lipinski et al. 2001). Lipinski and later Veber (Veber et al. 2002) suggested “rules of thumb” to be followed by medicinal chemists, concerning the accepted margins of property values (nHBDon < 5, nHBAcc < 10, MW < 500, XlogP < 5, TPSA < 140 A^2^) that ensure oral bioavailability. Indeed, across the two methods (ANN and single decision tree) used to model *logPe*, the topological descriptor tpsaEfficiency—representing the polar surface area of a molecule expressed as a ratio to molecular size—ranked first on the list of features evaluated as most relevant (Supporting Information1, S1.5). In the decision tree, tpsaEfficiency is depicted as the root node, as well as the second and third node (Fig. 11). The list of high ranking descriptors invariably included—although in different order depending on the method selected—features related to lipophilicity (octanol/water partition coefficients XlogP and AlogP) and the number of hydrogen bond donors in a molecule (nHBDon). A clear path in the decision tree indicated by the nodes 1, 2, 5, 10, 11 and 22 suggests that for acidic compounds (nAcid >  = 0.44)—shown to have “lower permeability”—charge is influential, whereas for the non-acidic molecules (nAcid <  = 0.44) lipophilicity and the number of hydrogen bond donors are decisive. The results from both modelling methods suggesting that non-polar, lipophilic and uncharged molecules are more likely to penetrate the highly hydrophobic intestinal cell membranes chime with previous reports (Chi et al. 2019; Oja and Maran 2015a, b, c, 2016a, b, 2018). However, given the pH variation in the intestinal environment (Avdeef 2001), measuring membrane permeability at only neutral pH (7.4) may eliminate compounds with good absorption characteristics at other pH values (Oja and Maran 2015a, 2015b, 2016a). Acidic compounds like valsartan or salicylic acid (IDs 186 and 144, Supporting Information 1, S1.1) show good permeability in acidic pH (5) only (logPe values – 5.99 and –5.53, respectively) (Oja and Maran 2015b).

### Importance of descriptors

**Fig. 12:**
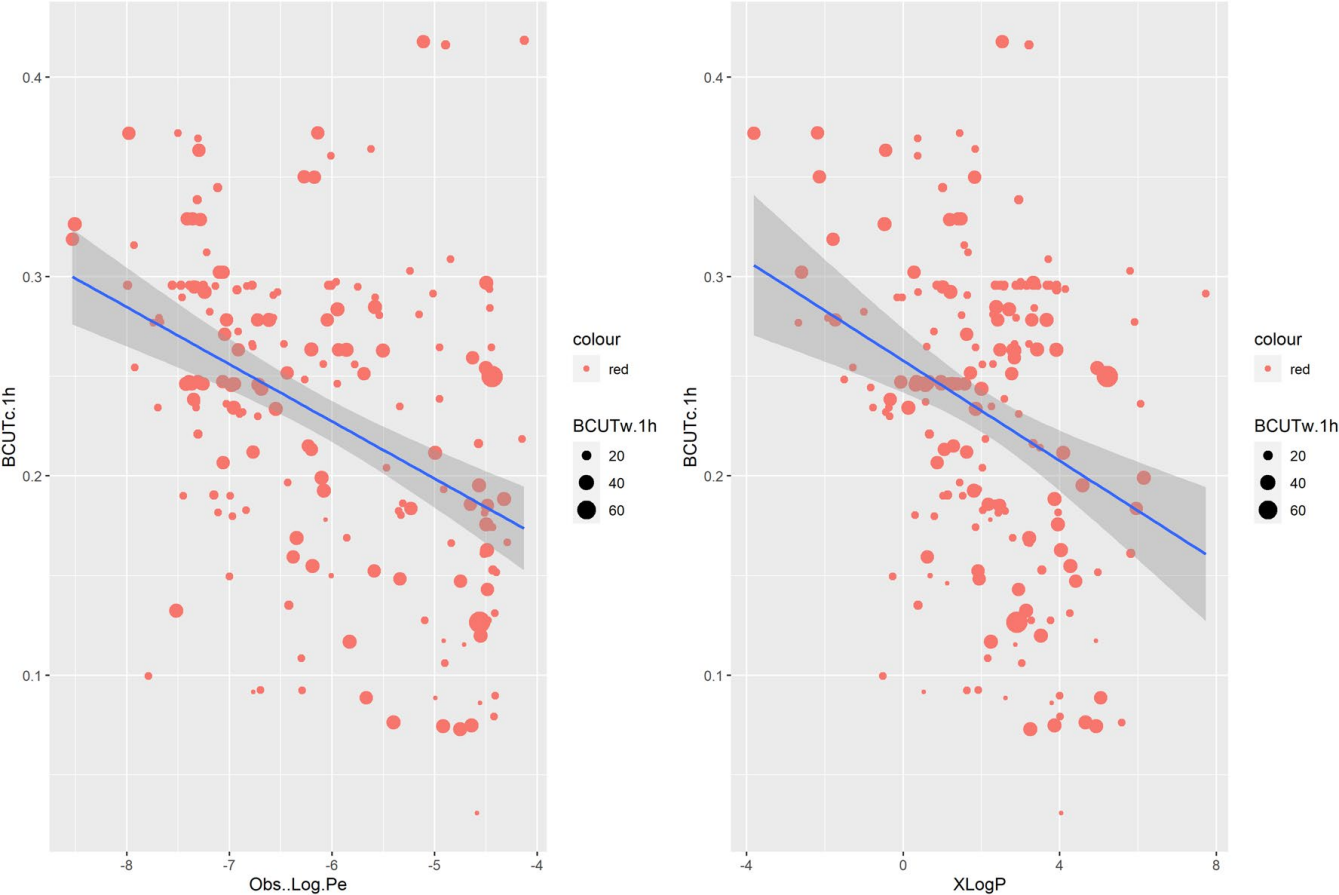
The negative relationship between BCUTc1h and *LogPe* (permeability) as well as between BCUTc1 and XLogP (lipophilicity) is presented. In each scatterplot, the dots are sized according to a third variable, i.e. the structural descriptor BCUTw1h. It can be observed that more than one structural combinations could lead to the same *LogPe* and *XlogP* values

Unlike previous attempts mostly depending on the combination of few “Lipinski-like” properties to explain the permeability of molecules, the present work aims to highlight the importance of additionally using meaningful structural information in modelling *LogPe*. Indeed, in Sect. 3.3 of the manuscript, we have shown that a set of six structural descriptors could capture approximately 41% of the variation in our data. Most interestingly, the BCUT descriptors are previously reported to allow for reversible decoding (Masek et al. 2008). While the combination of Lipinski-like descriptors may carry sufficient information to deduce substructural features, the high precision coordinates in BCUTs reveal a high level of detail from which a unique chemical structure or closely related analogues can be derived (Masek et al. 2008). The BCUT metrics introduced by Pearlman (Pearlman and Smith 2022) are whole-molecule descriptors that combine two or more measures of atom-based properties into a single value and are significant in measuring molecular diversity (Stanton 1999). Being extensions of previously developed parameters (Burden 1989), they further expand the number and types of atomic features that can be considered and provide a greater variety of proximity measures and weighting (Stanton 1999). Currently, three weighting schemes are employed: atomic weight (BCUTw), partial charge (BCUTc) and polarizability (BCUTp). In Fig. 12 the negative relationship between BCUTc1h and *LogPe* ( permeability) as well as between BCUTc1 and *XLogP* (lipophilicity) is presented.

Each dot on both sides of the line represents an observation, i.e. a molecule with an observed *logPe* and a calculated BCUTc1h value. In each scatterplot, the dots are sized according to a third variable, i.e. the structural descriptor BCUTw1h. It can be observed that more than one structural combinations could lead to the same *LogPe* and *XlogP* values.

**Fig. 13:**
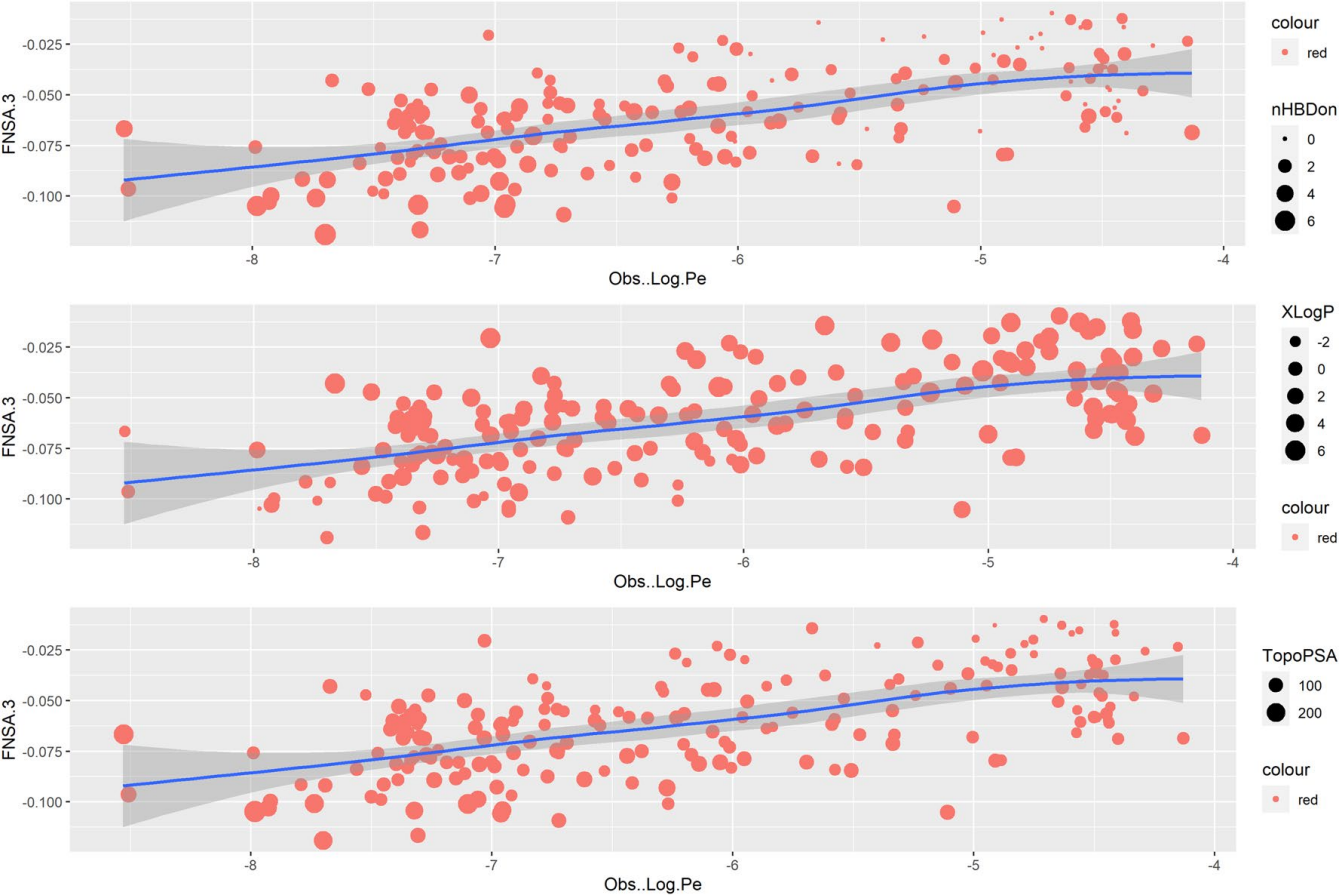
Illustration of the relationship between the descriptor FNSA.3 and the observed *LogPe.* Each dot on both sides of the line represents an observation, i.e. a molecule with an observed *logPe* and a calculated FNSA.3 value. The overall pattern of the graph suggests that higher FNSA.3 values are generally associated with increased permeability (approximately logPe ≥ -− 6.2). In each scatterplot, the dots are sized according to a third variable, i.e. the descriptors nHBDon, XlogP and TopoPSA (topological polar surface area), respectively, to explore their influence on the observed permeability. It can be clearly seen that an increase of FNSA.3 combined with low nHBDon and TopoPSA values and high XlogP (> 0, < 6) result in increased permeability

The geometric descriptor FNSA.3, which combines the surface area and partial charge information (charge weighted partial negative surface area/total molecular surface area) was estimated by the ANN models as highly significant, which is in accord with previous reports (14–19). In Fig. 5, where the correlation of the top 6 out of 61 most important descriptors along with the modelled end point *logPe* is presented, we can see that a positive linear association is observed between the descriptor FNSA.3 and *logPe*. This is better shown in Fig. 13, where a general illustration of the relationship between the two variables is provided. Each dot on both sides of the line represents an observation, i.e. a molecule with an observed *logPe* and a calculated FNSA.3 value. The overall pattern of the graph suggests that higher FNSA.3 values are generally associated with increased permeability (approximately *logPe* ≥ -6.2). In each scatterplot, the dots are sized according to a third variable, i.e. the descriptors nHBDon, XlogP and TopoPSA (topological polar surface area), respectively, to explore their influence on the observed permeability. It can be clearly seen that an increase of FNSA.3 combined with low nHBDon and TopoPSA values and high XlogP (> 0, < 6) result in increased permeability.

In Table 6, structures of the ten best performing molecules (out of the 1561, PostEra) in terms of their activity against M^pro^ (r_avg_IC_50_ < 1uM) are presented, along with their *logPe* as predicted by the model *EnsembleNN*. The “Lipinski-like” properties of the synthesized compounds are compliant with the rule of five and high permeability is predicted for the majority of them. Electron-withdrawing sulphonyl functional groups in molecules 1145 and 1245 appear to have negative effect on their membrane permeability. Although the presence of sulphonyl groups increases the number of hydrogen bond acceptors and enhance the binding affinity of drugs with target proteins through hydrogen bond interactions, they also increase polarity and affect solubility and acid–base properties (Fei et al. 2016), features already shown to be highly relevant for the membrane permeability of molecules.

**Table 6:**
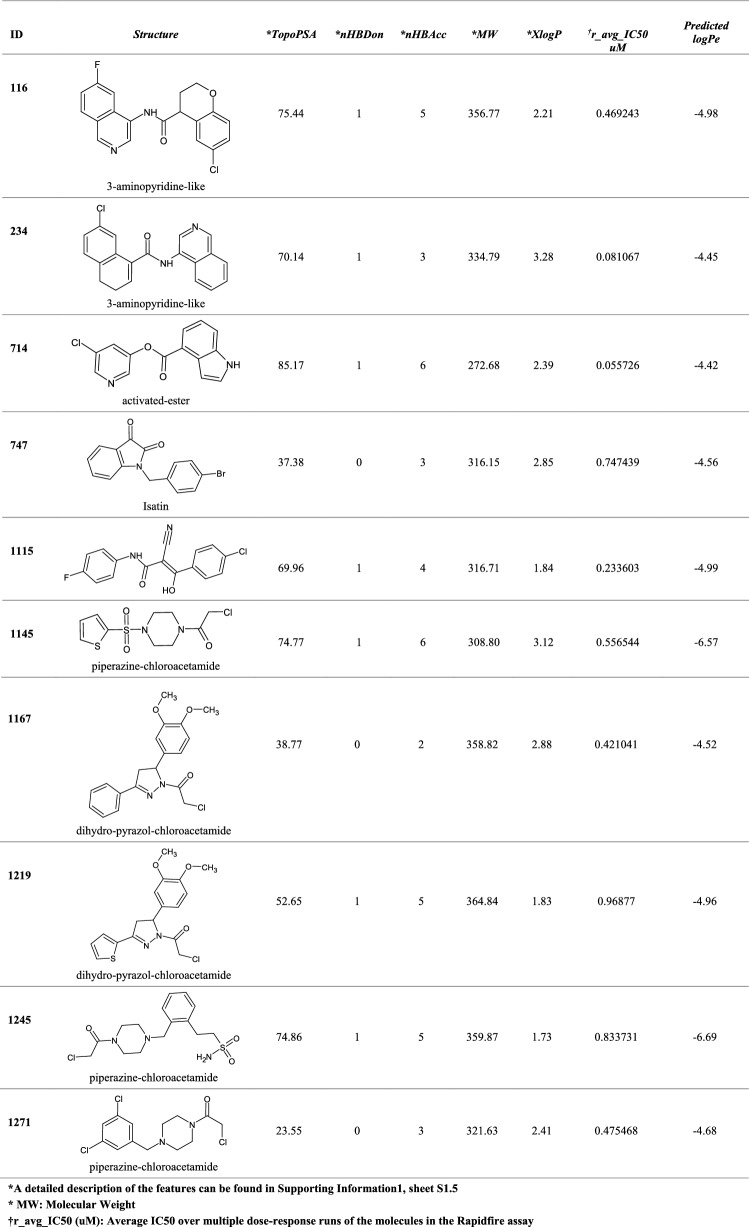
Structures of the ten best performing molecules (out of the 1561) in terms of their activity against the main protease M^pro^ (r_avg_IC50 < 1uM) are presented, along with their logPe as predicted by the model EnsembleNN

## Conclusions

In the present work, we employed a “stacked regression” ensemble approach to model the *effective membrane permeability coefficient LogPe* of 190 compounds, measured by the PAMPA assay at pH 7.4. On the whole, built on the set of 61 selected descriptors, our ensemble ANN model provides a method for the quantification of drug-likeness, particularly useful for drugs that move beyond the traditional rule of five space to access challenging targets (Alex et al. 2011). Unlike Lipinski’s rule, used for filtering large datasets of molecules and achieving no other discrimination of compounds beyond a qualitative pass or fail, our model allows for the optimization of relevant physicochemical properties of new molecules of interest—even before they are synthesized—through careful multi-criteria evaluation and control.

## Data, software and code availability

Publicly available permeability data (Chi et al. 2019) containing the SMILES strings of 190 structurally diverse drug or drug-like molecules with recorded effective permeability (logPe) values were used for creating the QSAR models in the present work. The data—carefully curated by Chi et al. (Chi et al. 2019) and based on previous reports by Oja et Maran (2015a, b, c, 2016a, b, 2018)—were generated with the same experimental protocol and were therefore highly homogenous. A single 3D conformation was created for each structure using the publicly available Bioclipse software (Spjuth et al. 2007, 2009). Data analysis and QSAR modelling were performed using the publicly available R Statistical Programming Language (version 3.5.1, 64bit and 4.0.3, 64BIT) (R Core Team 2018). R is both a language and an environment for statistical computing and graphics, providing a wide variety of statistical (linear and nonlinear modelling, classical statistical tests, time-series analysis, classification, clustering) and graphical techniques and is highly extensible. R is designed around a true computer language allowing users to add additional functionality by defining new functions. Extended functionalities are added to R by installing a number of packages, including Machine Learning algorithms implemented as third party libraries. The following R packages were used for the analysis: rcdk (Guha 2007), randomForest (Liaw and Wiener 2002), caret (Kuhn 2019, 2008), rpart (Therneau and Atkinson 2018), rpart.plot (Milborrow 2019), caretEnsemble (Deane-Mayer and Knowles 2016), tidyverse (Wickham et al. 2019a), mlbench (Leisch and Dimitriadou 2010), corrplot (Wei and Simko 2017), neuralnet (Günther et al. 2010), and dplyr (Wickham et al. 2019b), magrittr (Bache and Wickham 2014), WVPlots (Mount and Zumel 2020). The R code, a file detailing the versions of all R packages and individual subsets saved as CSV files for reading into the R modelling workflows have been made available on Zenodo (Gousiadou 2021), along with a README file explaining their contents and guidance on how to reproduce results via running the available code files.

The model has been implemented as a web service in the Jaqpot 5 modelling platform (Sarimveis 2019; ) and is available at the following URL: https://app.jaqpot.org/model/NDmy6udCm9UjmEMq0Zaq under the NanoCommons organization. In the “overview” tab, details about the model are presented. For accessing the model, the interested user should first register in Jaqpot 5 and then become a member of the NanoCommons organization by sending an e-mail to: E-mail: hsarimv@central.ntua.gr.

## Supporting information

Supplementary material for this work is included in the Supporting Information 1, as different sheets of an Excel workbook (also including the SMILES strings of all the compounds) and in the Supporting Information 2 where the structures of 47 most bioactive molecules (out of 1561 downloaded from the PostEra site with recorded bioactivity against M^pro^) along with their recorded IC_50_ and predicted *LogPe* values are depicted.

## Supplementary Information

Below is the link to the electronic supplementary material.

Supplementary file1 (XLSX 1056 kb)

Supplementary file2 (DOCX 274 kb)

## Data Availability

The data that support the findings of this study are openly available in Zenodo Online Repository https://zenodo.org/record/5504324#.Y5sPp3bMJaQ.
