# Supplementary material for: Development of artificial neural network models to predict the PAMPA effective permeability of new, orally administered drugs active against the coronavirus SARS-CoV-2
Source: Netw Model Anal Health Inform Bioinform. 2023 Feb 6;12(1):16. doi: 10.1007/s13721-023-00410-9 (PMC9901841; doi:10.1007/s13721-023-00410-9)
Supplement: Supplementary file 2 — Supplementary file2 (DOCX 274 kb) [file 13721_2023_410_MOESM2_ESM.docx]

| *Supporting Information 2: A table depicting the structures of 47 most bioactive molecules (out of 1561 downloaded from PostEra with recorded bioactivity against M^pro^) along with their recorded IC50 and predicted LogPe values.* | | | | |
| --- | --- | --- | --- | --- |
| *ID* | *SMILES & STRUCTURE* | *r_avg_IC50 uM** | *f_avg_IC50 uM** | *EnsembleNN- Predicted logPe* |
| 114 | **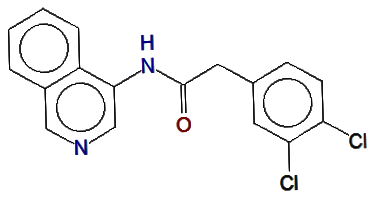O=C(Cc1ccc(Cl)c(Cl)c1)Nc1cncc2ccccc12** | **0.131608** | **0.261213** | **-4.27** |
| 116 | **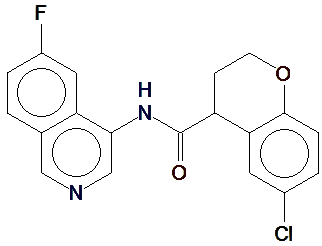O=C(Nc1cncc2ccc(F)cc12)C1CCOc2ccc(Cl)cc21** | **0.469243** | **0.766616** | **-4.98** |
| 153 | **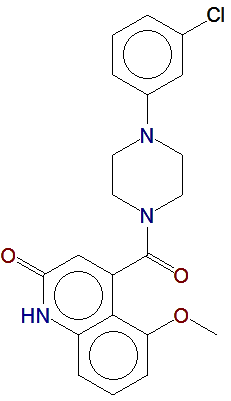COc1cccc2[nH]c(=O)cc(C(=O)N3CCN(c4cccc(Cl)c4)CC3)c12** | **1.844528** | **4.684288** | **-4.45** |
| 170 | **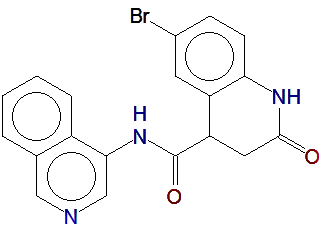O=C1CC(C(=O)Nc2cncc3ccccc23)c2cc(Br)ccc2N1** | **1.121994** | **1.122878** | **-4.54** |
| 234 | **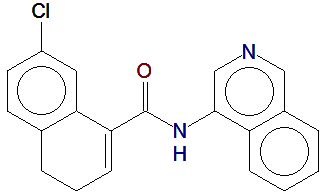O=C(Nc1cncc2ccccc12)C1=CCCc2ccc(Cl)cc21** | **0.081067** | **0.233267** | **-4.45** |
| 255 | **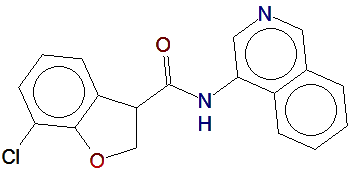O=C(Nc1cncc2ccccc12)C1COc2c(Cl)cccc21** | **5.359335** | **4.757496** | **-4.35** |
| 288 | **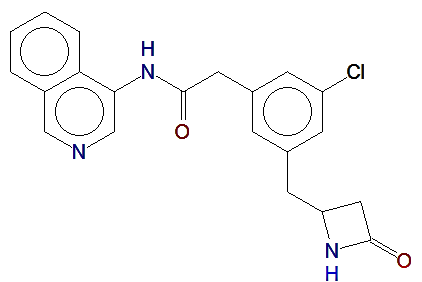O=C(Cc1cc(Cl)cc(CC2CC(=O)N2)c1)Nc1cncc2ccccc12** | **4.42082** | **4.567055** | **-5.01** |
| 289 | **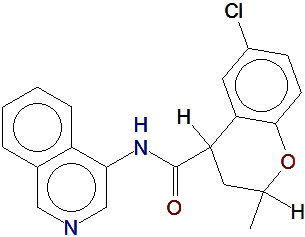C[C@@H]1C[C@H](C(=O)Nc2cncc3ccccc23)c2cc(Cl)ccc2O1** | **2.655065** | **0.67929** | **-4.47** |
| 293 | **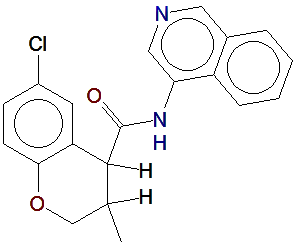C[C@@H]1COc2ccc(Cl)cc2[C@@H]1C(=O)Nc1cncc2ccccc12** | **1.829709** | **1.713488** | **-4.42** |
| 307 | **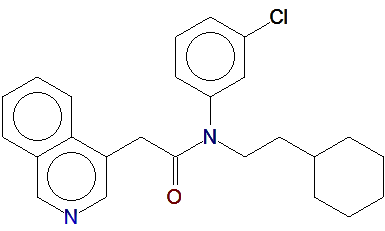O=C(Cc1cncc2ccccc12)N(CCC1CCCCC1)c1cccc(Cl)c1** | **1.021087** | **2.112459** | **-4.93** |
| 672 | **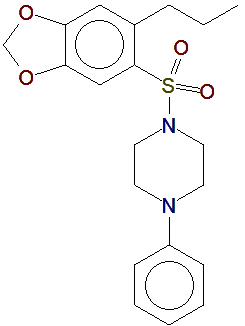CCCc1cc2c(cc1S(=O)(=O)N1CCN(c3ccccc3)CC1)OCO2** | **15.96943** | **n/a** | **-4.37** |
| 675 | **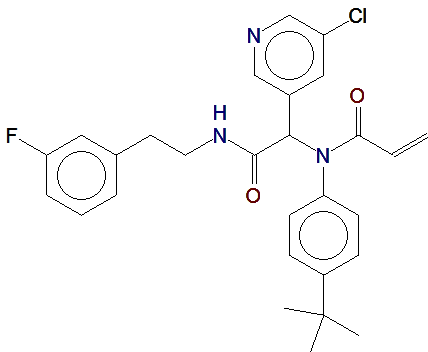C=CC(=O)N(c1ccc(C(C)(C)C)cc1)C(C(=O)NCCc1cccc(F)c1)c1cncc(Cl)c1** | **2.112801** | **11.47977** | **-4.54** |
| 683 | **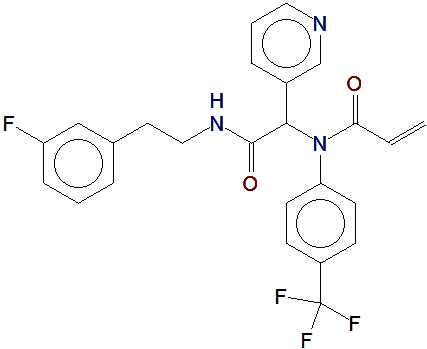C=CC(=O)N(c1ccc(C(F)(F)F)cc1)C(C(=O)NCCc1cccc(F)c1)c1cccnc1** | **6.116962** | **20.12027** | **-4.72** |
| 685 | **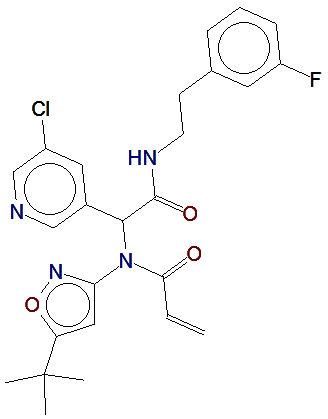C=CC(=O)N(c1cc(C(C)(C)C)on1)C(C(=O)NCCc1cccc(F)c1)c1cncc(Cl)c1** | **4.777124** | **n/a** | **-4.85** |
| 711 | **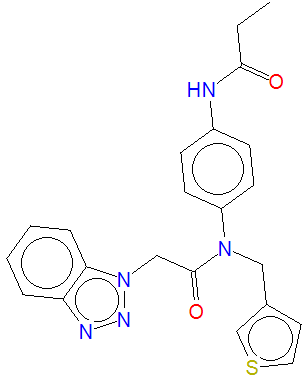CCC(=O)Nc1ccc(N(Cc2ccsc2)C(=O)Cn2nnc3ccccc32)cc1** | **12.56226** | **1.627637** | **-5.76** |
| 714 | **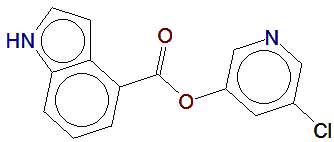O=C(Oc1cncc(Cl)c1)c1cccc2[nH]ccc12** | **0.055726** | **0.05** | **-4.42** |
| 734 | **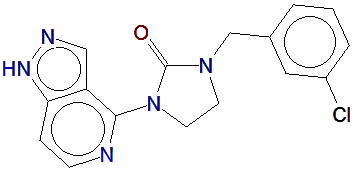O=C1N(Cc2cccc(Cl)c2)CCN1c1nccc2[nH]ncc12** | **2.907617** | **4.557674** | **-5.00** |
| 736 | **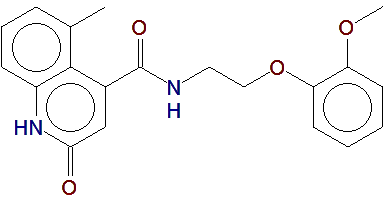COc1ccccc1OCCNC(=O)c1cc(=O)[nH]c2cccc(C)c12** | **2.601503** | **10.62102** | **-5.39** |
| 747 | **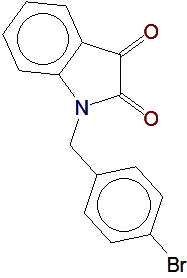**  **O=C1C(=O)N(Cc2ccc(Br)cc2)c2ccccc21** | **0.747439** | **198** | **-4.56** |
| 830 | **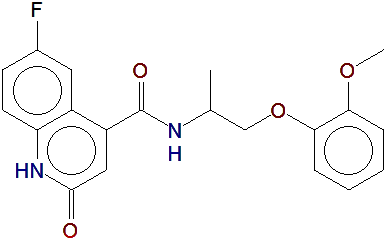COc1ccccc1OCC(C)NC(=O)c1cc(=O)[nH]c2ccc(F)cc12** | **27.47431** | **16.44139** | **-5.71** |
| 909 | **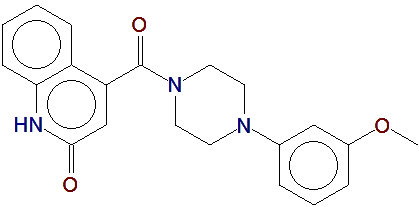COc1cccc(N2CCN(C(=O)c3cc(=O)[nH]c4ccccc34)CC2)c1** | **19.69977** | **7.54963** | **-4.36** |
| 929 | **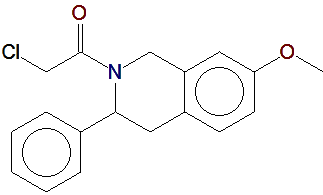COc1ccc2c(c1)CN(C(=O)CCl)C(c1ccccc1)C2** | **1.784272** | **3.739105** | **-4.31** |
| 938 | **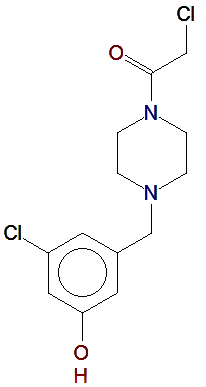**  **O=C(CCl)N1CCN(Cc2cc(O)cc(Cl)c2)CC1** | **1.568926** | **12.91845** | **-4.77** |
| 959 | **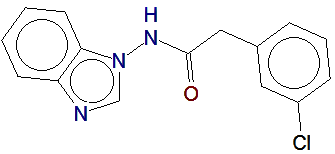O=C(Cc1cccc(Cl)c1)Nn1cnc2ccccc21** | **4.265198** | **8.303468** | **-4.60** |
| 976 | **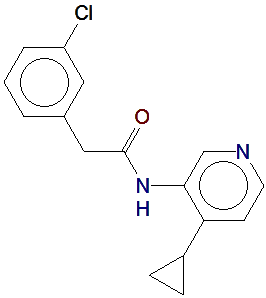O=C(Cc1cccc(Cl)c1)Nc1cnccc1C1CC1** | **7.397974** | **7.173636** | **-4.74** |
| 1034 | **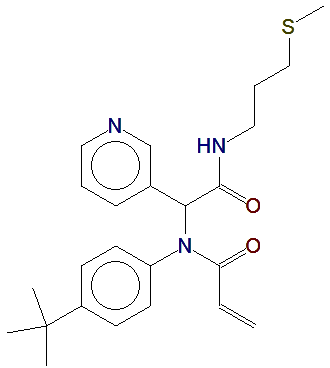C=CC(=O)N(c1ccc(C(C)(C)C)cc1)C(C(=O)NCCCSC)c1cccnc1** | **13.68893** | **25.46808** | **-5.59** |
| 1041 | **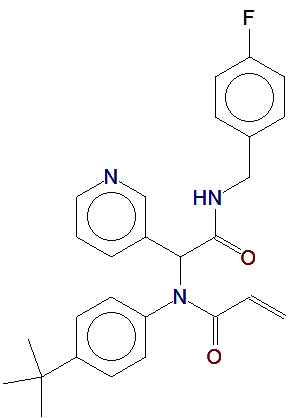C=CC(=O)N(c1ccc(C(C)(C)C)cc1)C(C(=O)NCc1ccc(F)cc1)c1cccnc1** | **10.50654** | **23.33478** | **-4.63** |
| 1056 | **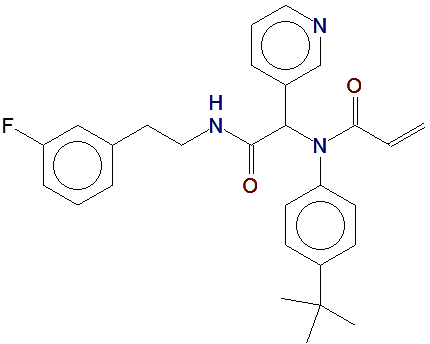C=CC(=O)N(c1ccc(C(C)(C)C)cc1)C(C(=O)NCCc1cccc(F)c1)c1cccnc1** | **3.358824** | **2.958399** | **-4.62** |
| 1115 | **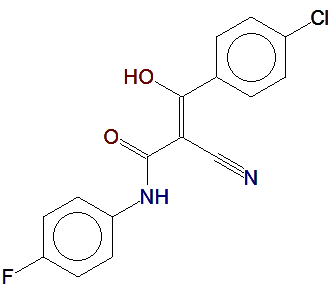N#C/C(C(=O)Nc1ccc(F)cc1)=C(/O)c1ccc(Cl)cc1** | **0.233603** | **n/a** | **-4.99** |
| 1143 | **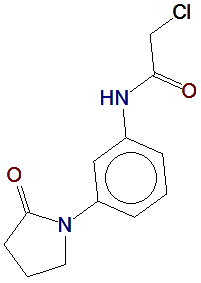**  **O=C(CCl)Nc1cccc(N2CCCC2=O)c1** | **n/a** | **70.32858** | **-6.04** |
| 1144 | **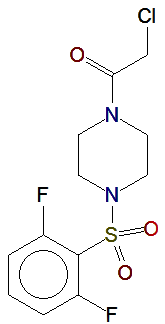**  **O=C(CCl)N1CCN(S(=O)(=O)c2c(F)cccc2F)CC1** | **2.623214** | **3.284511** | **-6.76** |
| 1145 | **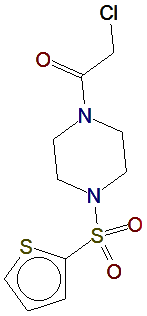**  **O=C(CCl)N1CCN(S(=O)(=O)c2cccs2)CC1** | **0.556544** | **2.161829** | **-6.57** |
| 1146 | **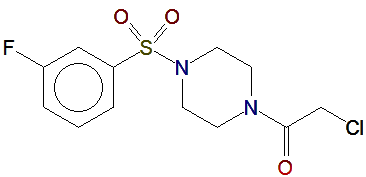O=C(CCl)N1CCN(S(=O)(=O)c2cccc(F)c2)CC1** | **2.023561** | **4.550489** | **-6.18** |
| 1148 | **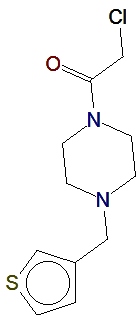**  **O=C(CCl)N1CCN(Cc2ccsc2)CC1** | **1.137302** | **3.49772** | **-4.71** |
| 1162 | **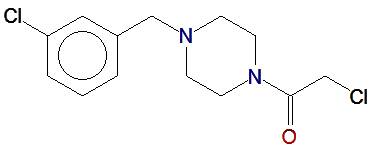O=C(CCl)N1CCN(Cc2cccc(Cl)c2)CC1** | **0.545448** | **3.137414** | **-4.57** |
| 1167 | **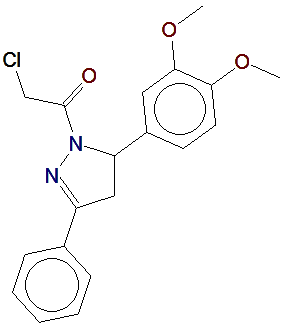COc1ccc(C2CC(c3ccccc3)=NN2C(=O)CCl)cc1OC** | **0.421041** | **0.542597** | **-4.52** |
| 1170 | **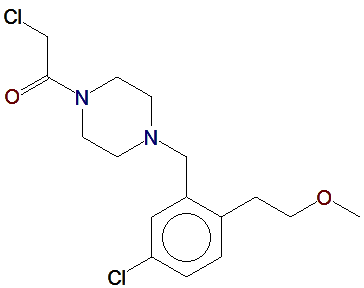COCCc1ccc(Cl)cc1CN1CCN(C(=O)CCl)CC1** | **2.417464** | **2.202279** | **-4.80** |
| 1219 | **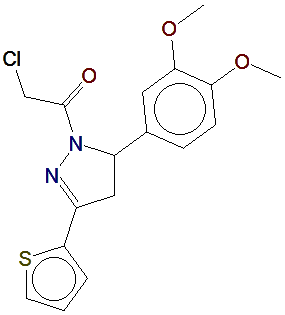COc1ccc(C2CC(c3cccs3)=NN2C(=O)CCl)cc1OC** | **0.96877** | **1.871183** | **-4.96** |
| 1222 | **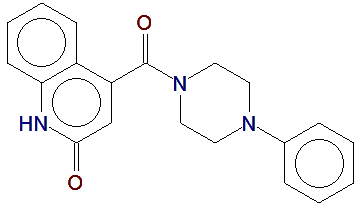O=C(c1cc(=O)[nH]c2ccccc12)N1CCN(c2ccccc2)CC1** | **10.40272** | **27.5249** | **-5.06** |
| 1245 | **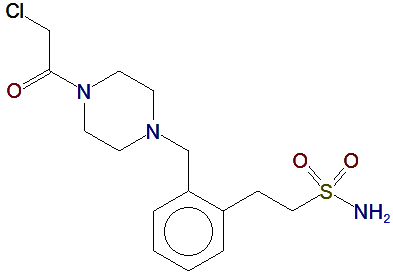NS(=O)(=O)CCc1ccccc1CN1CCN(C(=O)CCl)CC1** | **0.833731** | **2.717021** | **-6.69** |
| 1271 | **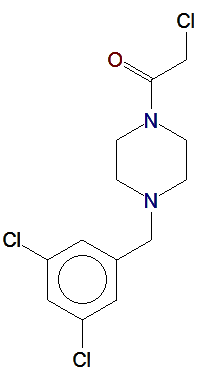**  **O=C(CCl)N1CCN(Cc2cc(Cl)cc(Cl)c2)CC1** | **0.475468** | **1.889434** | **-4.68** |
| 1279 | **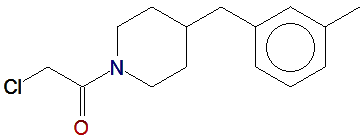Cc1cccc(CC2CCN(C(=O)CCl)CC2)c1** | **4.493787** | **14.72475** | **-4.27** |
| 1283 | **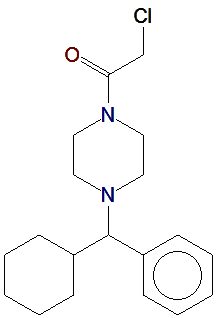O=C(CCl)N1CCN(C(c2ccccc2)C2CCCCC2)CC1** | **0.643563** | **4.128422** | **-4.88** |
| 1457 | **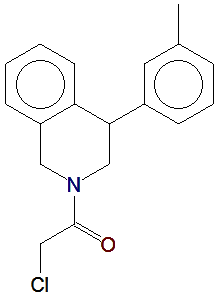**  **Cc1cccc(C2CN(C(=O)CCl)Cc3ccccc32)c1** | **0.419791** | **2.004125** | **-4.57** |
| 1511 | **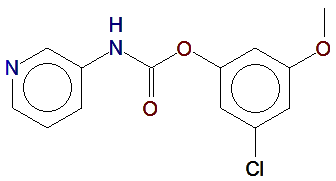COc1cc(Cl)cc(OC(=O)Nc2cccnc2)c1** | **1.51349** | **6.68442** | **-5.20** |
| 1523 | **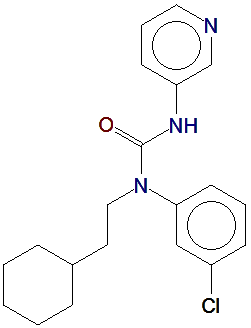O=C(Nc1cccnc1)N(CCC1CCCCC1)c1cccc(Cl)c1** | **3.300357** | **3.056672** | **-4.66** |
| 1566 | **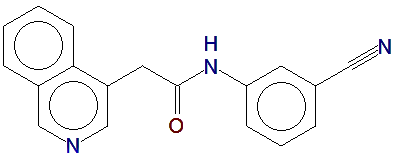N#Cc1cccc(NC(=O)Cc2cncc3ccccc23)c1** | **14.64109** | **26.68013** | **-6.82** |

*** r_avg_IC50: Average IC50 over the multiple dose-response runs of the molecule in the Rapidfire assay**

***f_avg_IC50: Average IC50 over the multiple dose-response runs of the molecule in the Fluorescence assay**
